# Supplementary material for: The disordered p53 transactivation domain is the target of FOXO4 and the senolytic compound FOXO4-DRI
Source: Nat Commun. 2025 Jul 1;16:5672. doi: 10.1038/s41467-025-60844-9 (PMC12216184; doi:10.1038/s41467-025-60844-9)
Supplement: Supplementary file 1 — Supplementary Information [file 41467_2025_60844_MOESM1_ESM.pdf]

Supplementary Information

The Disordered p53 Transactivation Domain is the Target of FOXO4 and the Senolytic Compound FOXO4-DRI

Benjamin Bourgeois*[1], Emil Spreitzer*[1], Daniel Platero-Rochart[1], Margret Paar[1], Qishun Zhou[1], Sinem Usluer[1], Peter L.J. de Keizer[2], Boudewijn M.T. Burgering[2], Pedro A. Sánchez-Murcia[1,3], Tobias Madl[1,3,#]

[1] Division of Medicinal Chemistry, Otto-Loewi Research Center, Medical University of Graz, Graz, Austria
[2] Department of Molecular Cancer Research, Center for Molecular Medicine, Division of Biomedical Genetics, University Medical Center Utrecht, Utrecht University, Universiteitsweg 100, 3584CG Utrecht, The Netherlands
[3] BioTechMed-Graz, 8010 Graz, Austria
*contributed equally
[#]Corresponding Author: tobias.madl@medunigraz.at

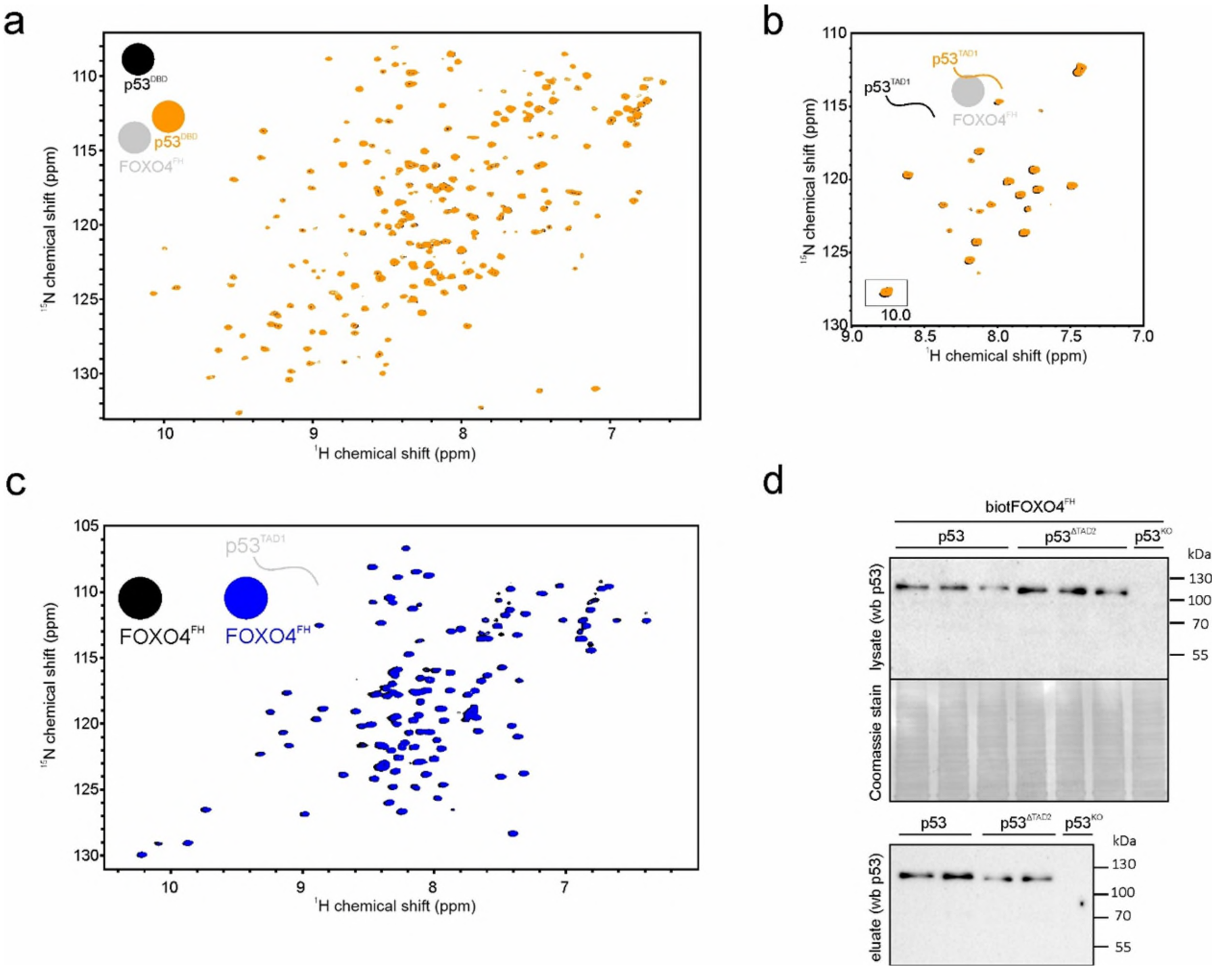

Supplementary Figure 1: p53^{TAD2} is the primary binding site of FOXO4^{FH}. (a) Overlay of ^{1}H,^{15}N HSQC spectra of ^{15}N-labeled p53^{DBD} at 100 µM in the absence (black) and presence (orange) of 100 µM FOXO4^{FH}. (b) Overlay of ^{1}H,^{15}N HSQC spectra of ^{15}N-labeled p53^{TAD1} at 100 µM in absence (black) and presence (bleu) of 100 µM FOXO4^{FH}. (c) Overlay of ^{1}H,^{15}N HSQC spectra of ^{15}N-labeled FOXO4^{FH} at 100 µM in absence (black) or presence (orange) of 100 µM p53^{TAD1}. (d) Pull down of endogenous p53, p53ΔTAD2 and p53KO with biotinylated biotFOXO^{FH} in RPE-1 cells.

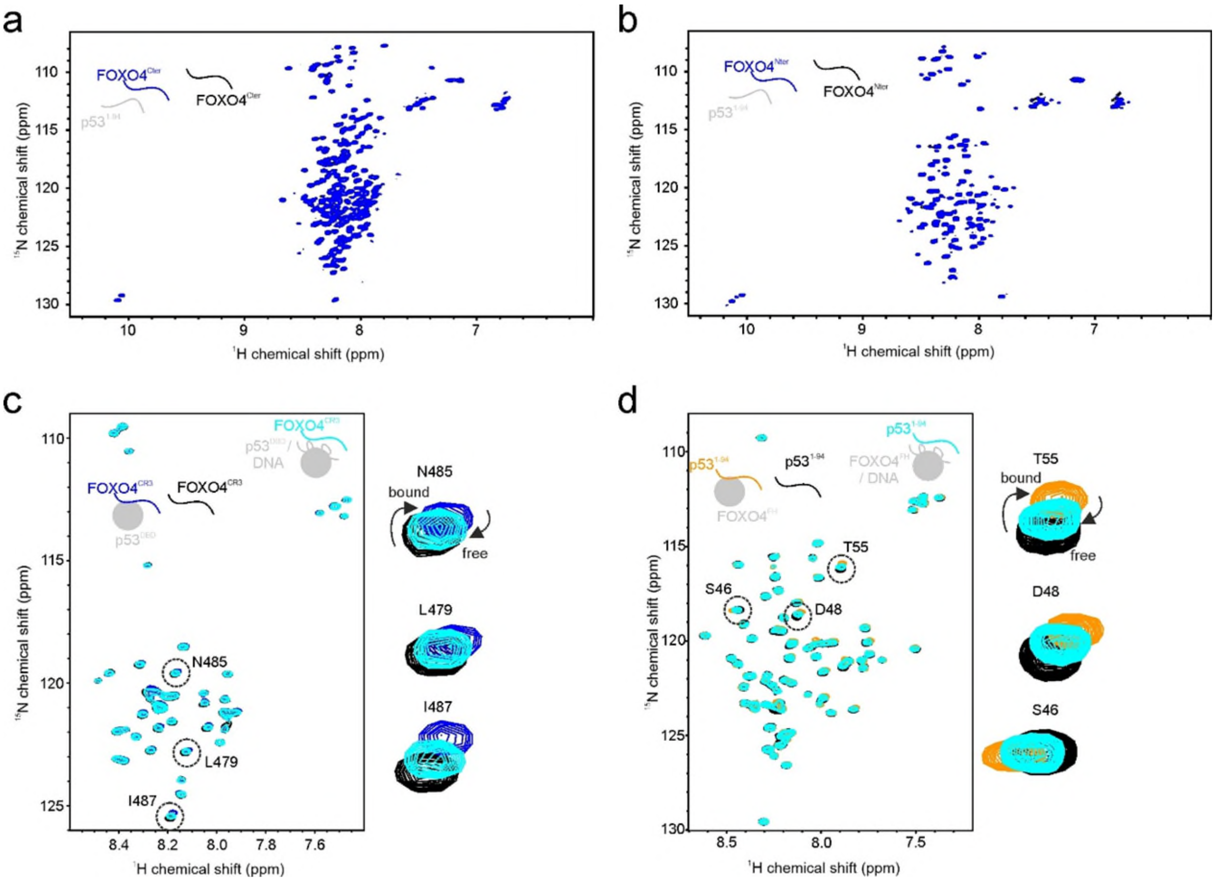

Supplementary Figure 2. Characterization of FOXO4 - p53 interactions: (a) ^{1}H,^{15}N HSQC spectrum of 100 µM ^{15}N-labeled FOXO4^{Cter} in the absence (black) or in presence of one stoichiometric equivalent of unlabeled p53$^{1\text{-}94}$ (blue). (b) ^{1}H,^{15}N HSQC spectrum of 100 µM ^{15}N-labeled FOXO4^{Nter} in the absence (black) or presence of one stoichiometric equivalent of unlabeled p53$^{1\text{-}94}$ (blue). (c) Overlay of 50 µM ^{15}N-labeled FOXO4^{CR3} in the absence (black) or presence of 200 µM p53^{DBD} (blue) or 50 µM CDKN1A-p53-RE and 200 µM p53^{DBD} (cyan). (d) Overlay of 50 µM ^{15}N-labeled p53$^{1\text{-}94}$ in the absence (black) or presence of 100 µM FOXO4^{FH} (orange) or 50 µM CTDSP2 and 100 µM FOXO4^{FH} (cyan).

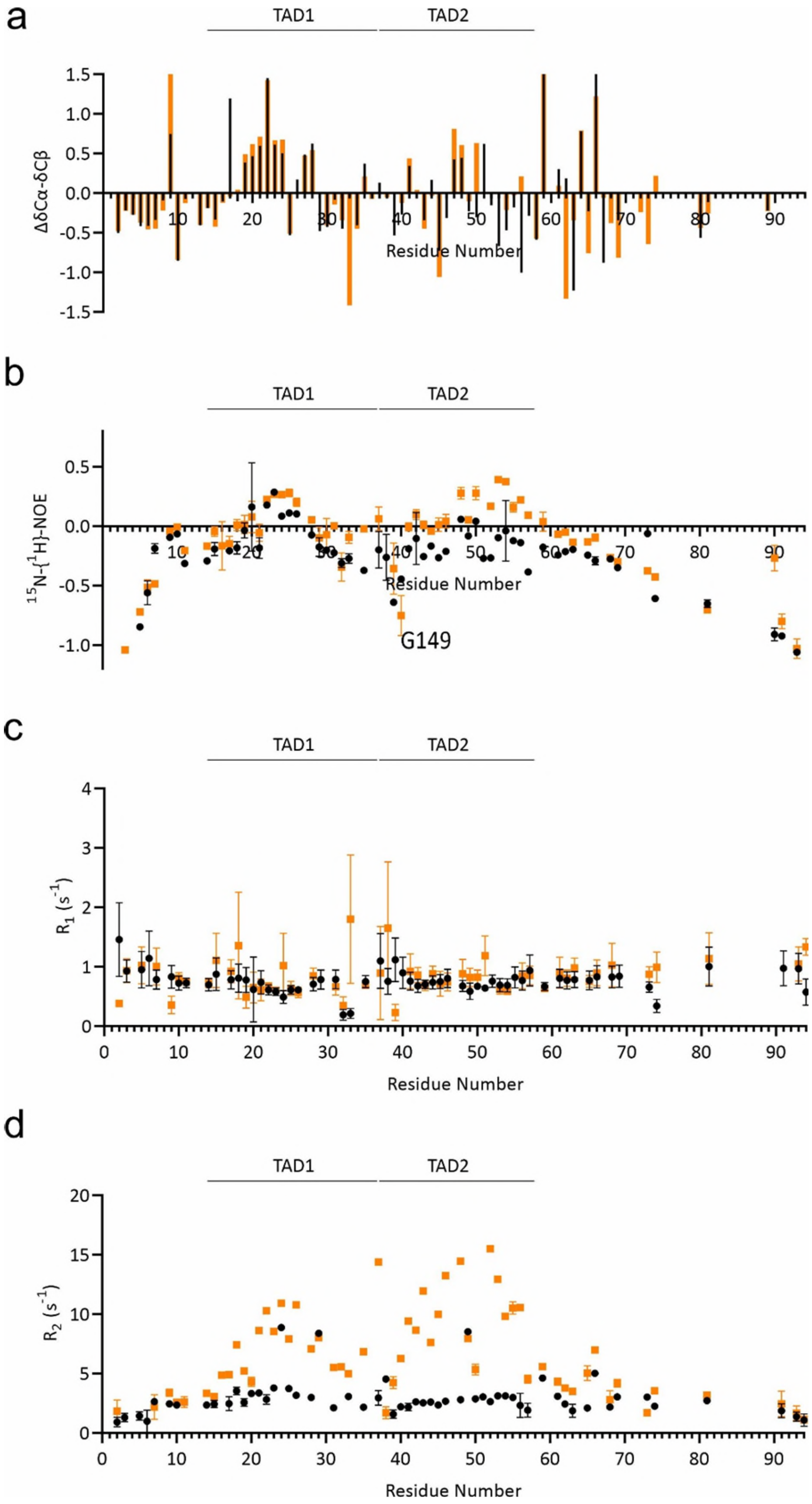

Supplementary Figure 3. FOXO4^{FH} binding enhances rigidity and secondary structure of p53^{TAD}: (a) Plot of secondary ^{13}Cα/^{13}Cβ chemical shifts of p53$^{1\text{-}94}$ in the absence (black) or presence of 400 μM unlabeled FOXO4^{FH} (orange). Secondary chemical shifts were obtained by subtracting the random coil chemical shifts (predicted by ncIDP) from observed chemical shifts. Differences between the secondary chemical shift deviations δCα − δCβ were plotted against the amino acid residue numbers, taking into account next neighbor effects. (b) ^{15}N{^{1}H} NOEs, (c) R_1 and R_2 (d) relaxation rates of 300 μM p53$^{1\text{-}94}$ in the absence (black) or presence of 400 μM unlabeled

FOXO4^{FH} (orange). Error bars in (b) (c) (d) were calculated based on the standard deviation of noise in the corresponding spectra and using error propagation.

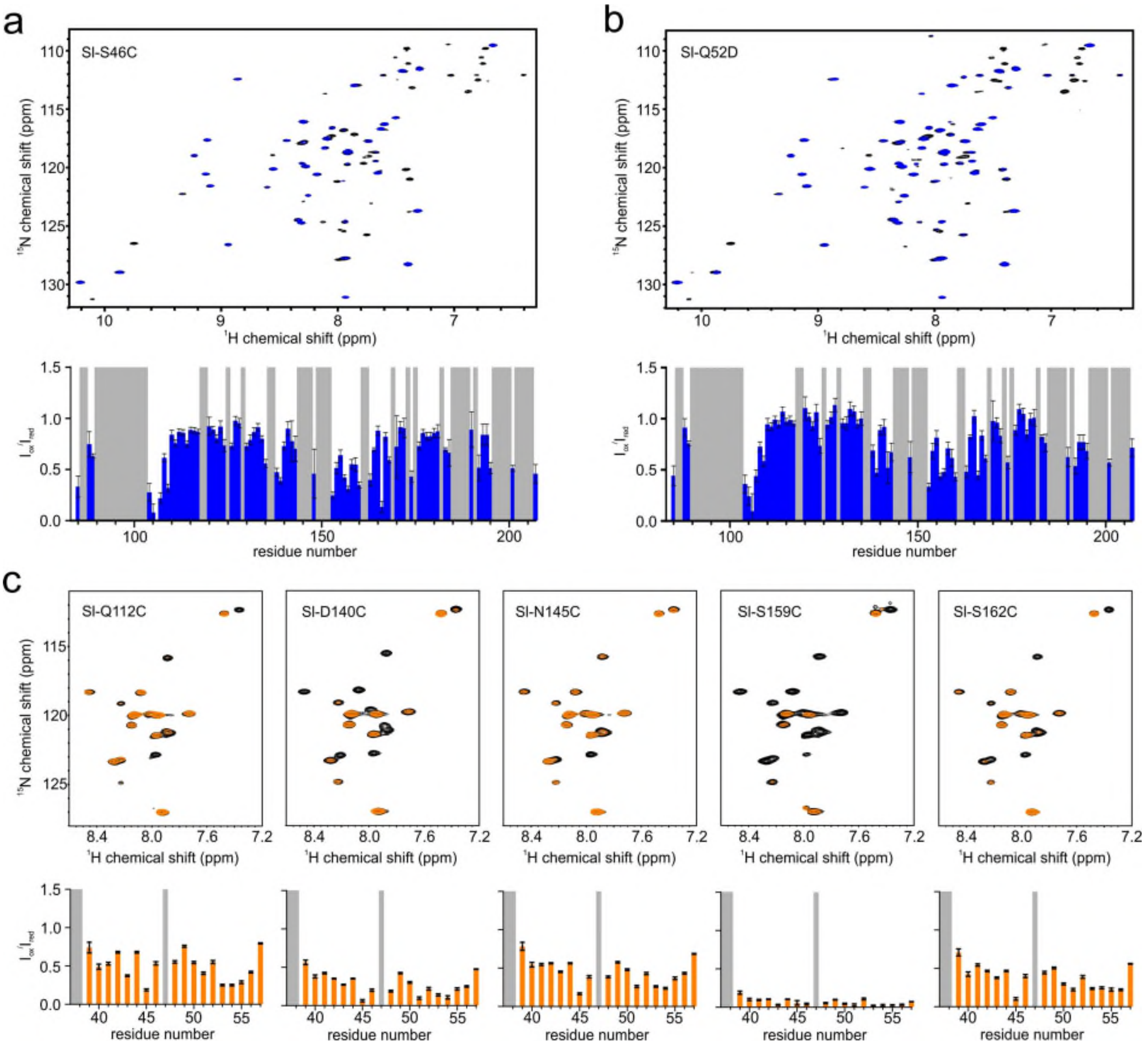

Supplementary Figure 4. PRE restraints used for the determination of the structure of FOXO4^{FH} – p53^{TAD2}. Paramagnetic relaxation enhancement (PRE) measurements of 100 μM ^{15}N-labeled FOXO4^{FH} in presence of p53^{TAD2} spin labeled at position 46 or 52 in (a) and (b), respectively. PRE data of 100 μM ^{15}N-labeled p53^{TAD2} in the presence of FOXO4^{FH} spin labeled at position 112, 140, 145, 159 and 162 in (c) The bar plots show the intensity ratio of the ^{1}H,^{15}N HSQC FOXO4^{FH} in (a, b) or p53^{TAD2} (c) obtained in the paramagnetic divided by the diamagnetic state. Error bars in (a) (b) and (c) were calculated based on the standard deviation of noise in the paramagnetic and diamagnetic spectra and using error propagation.

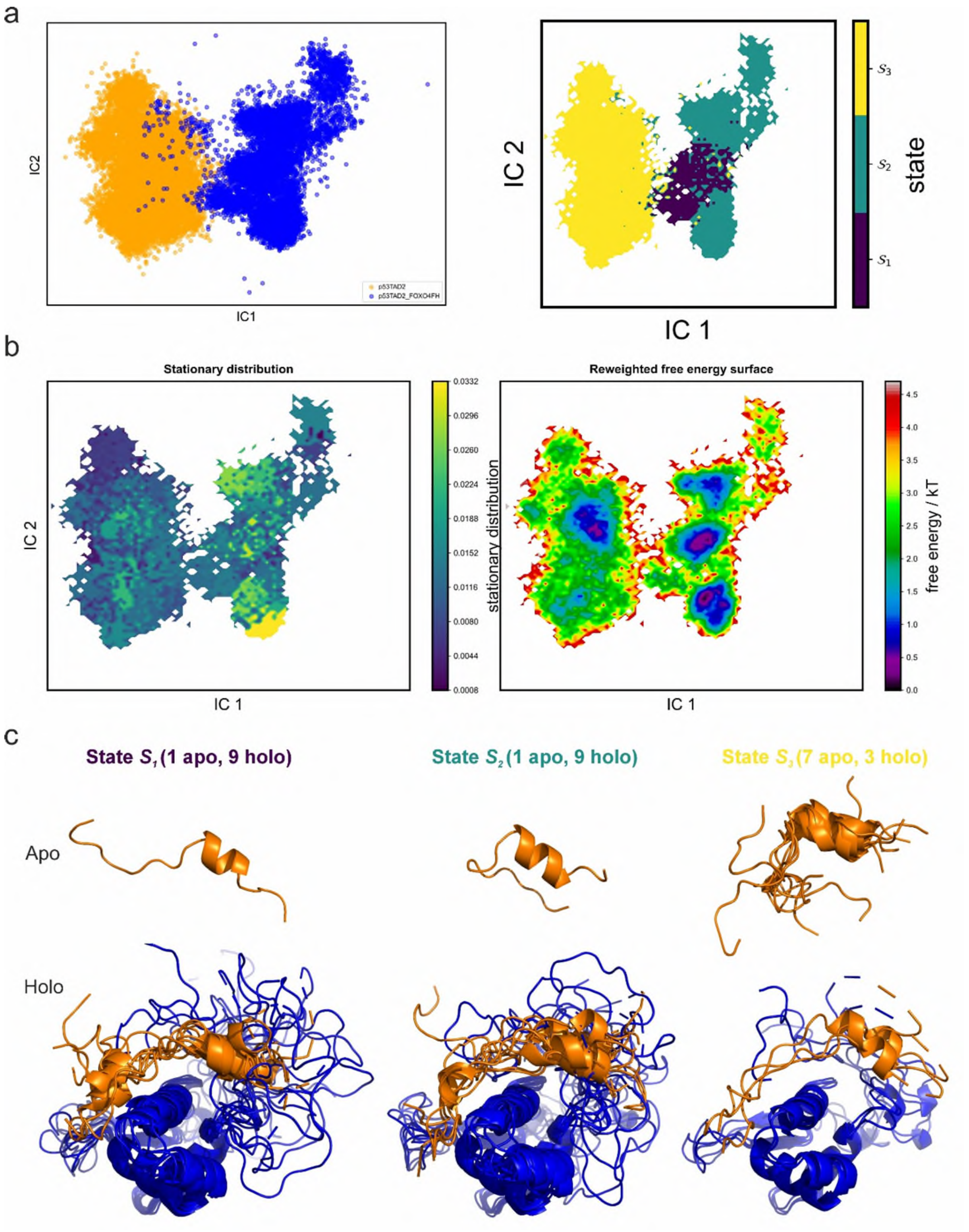

Supplementary Figure 5. Markov State Analysis of the p53^{TAD2}-FOXO4^{FH} complex. (a) Distribution of p53^{TAD2} microstates projected onto the first two time-lagged independent components (ICs) at lag time t=5 ns. The distribution of microstates was grouped and modeled into three distinct macrostates (S_1-S_3, right). (b) Stationary distribution and reweighted free energy surface of p53^{TAD2} projected onto the first two time-lagged independent components (ICs) at lag time t=5 ns. (c) Ensemble of representative structures of p53^{TAD2} (orange) bound to FOXO4^{FH} (dark blue) in the three macrostates displayed in (a).

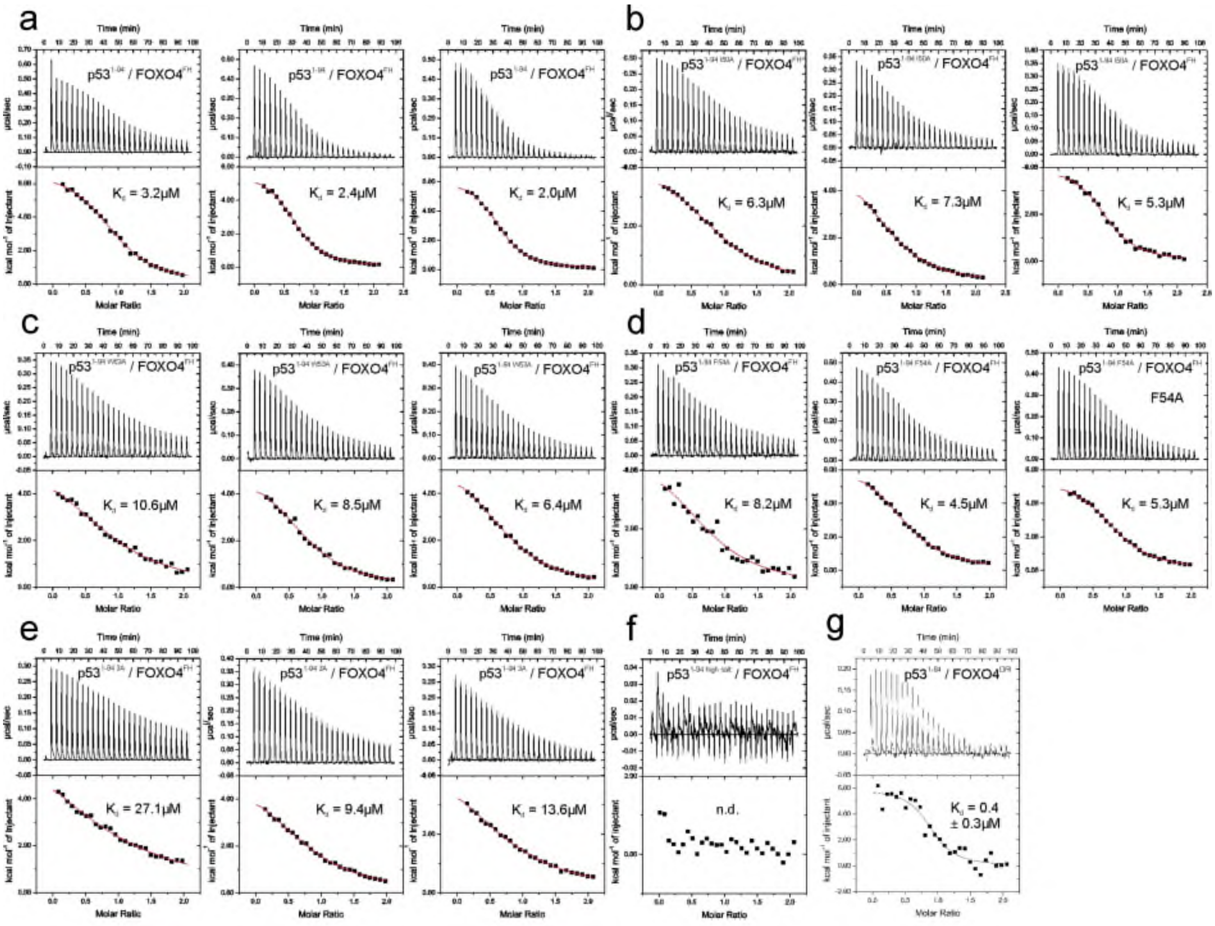

Supplementary Figure 6. $p53^{1\text{-}94}$ interacts with $FOXO4^{FH}$ via a network of hydrophobic and charge-based interactions: Titration of 25 µM of $p53^{1\text{-}94}$, $p53^{1\text{-}94\ I50A}$, $p53^{1\text{-}94\ W53A}$, $p53^{1\text{-}94\ F54A}$, $p53^{1\text{-}94\ 3A}$ and $p53^{1\text{-}94\ high\ salt}$ with 250 µM of $FOXO4^{FH}$ (a-f). Titration of 50 µM of $p53^{1\text{-}94}$ with 500 µM of $FOXO4^{DRI}$ in (g).

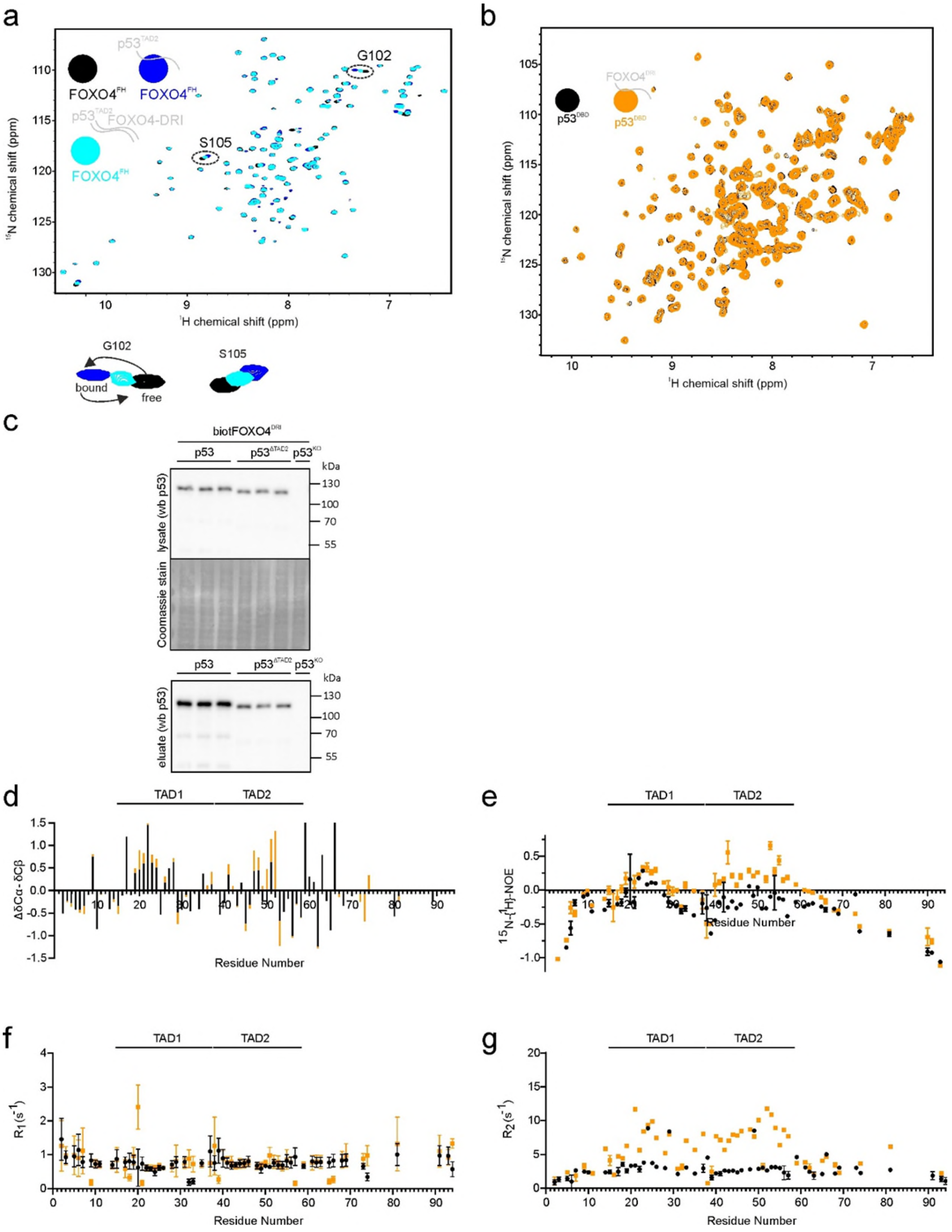

Supplementary Figure 7. Characterization of FOXO4-DRI binding. (a) Overlay of 100 µM ^{15}N-labeled FOXO4^{FH} in the absence (black) or presence of 200 µM p53^{TAD2} (blue) or 200 µM FOXO4-DRI and 200 µM p53^{TAD2} (cyan) (b) Overlay of 100 µM ^{15}N-labeled p53^{DBD} in the absence (black) or presence of 200 µM FOXO4-DRI (orange). (c) Pull down of endogenous p53, p53$^{\Delta TAD2}$ and p53^{KO} with biotinylated biotFOXO4^{DRI} in RPE-1 cells. (d) Plot of the ^{13}Cα/^{13}Cβ secondary chemical shifts of p53$^{1\text{-}94}$ in the absence (black) or presence of 400 µM unlabeled FOXO4-DRI (orange). (e) ^{15}N{^{1}H} NOEs of 300 µM p53$^{1\text{-}94}$ in the absence (black) or presence of 400 µM unlabeled FOXO4-DRI (orange). R_1 (f) and R_2 (g) relaxation rates of 300 µM p53$^{1\text{-}94}$ in the absence (black) or presence of 400 µM unlabeled FOXO4-DRI (orange). Error bars in (e) (f) (g) were calculated based on the standard deviation of noise in the corresponding spectra and using error propagation.

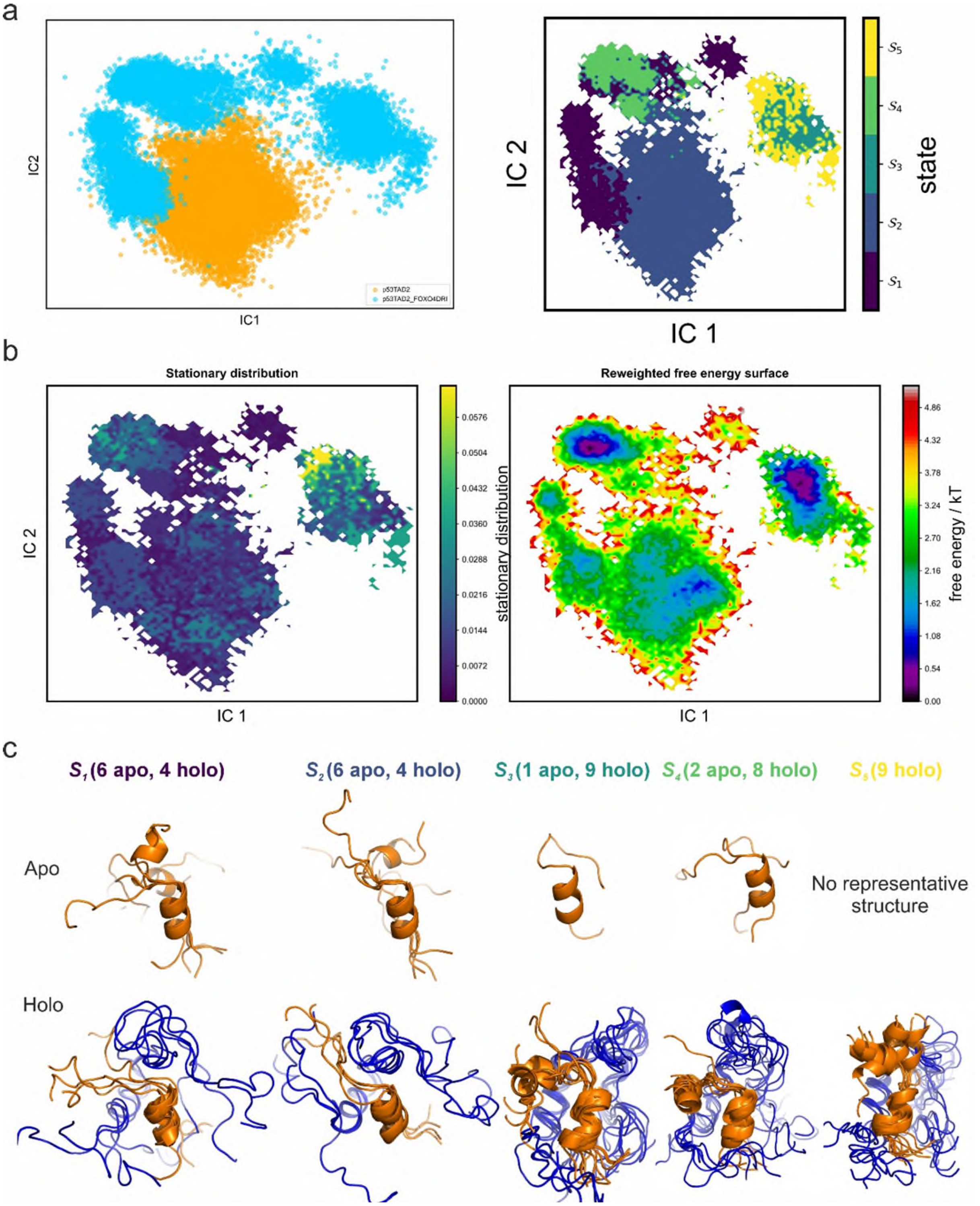

Supplementary Figure 8. Markov State Analysis of the $p53^{TAD2}$-FOXO4-DRI complex. (a) Distribution of $p53^{TAD2}$ microstates projected onto the first two time-lagged independent components (ICs) at lag time t=5 ns. The distribution of microstates was grouped and modeled into five distinct macrostates (S_1-S_5, right). (b) Stationary distribution and reweighted free energy surface of $p53^{TAD2}$ projected onto the first two time-lagged independent components (ICs) at lag time t=5 ns. (c) Ensemble of representative structures of $p53^{TAD2}$ (orange) bound to FOXO4-DRI (dark blue) in the three macrostates displayed in (a).

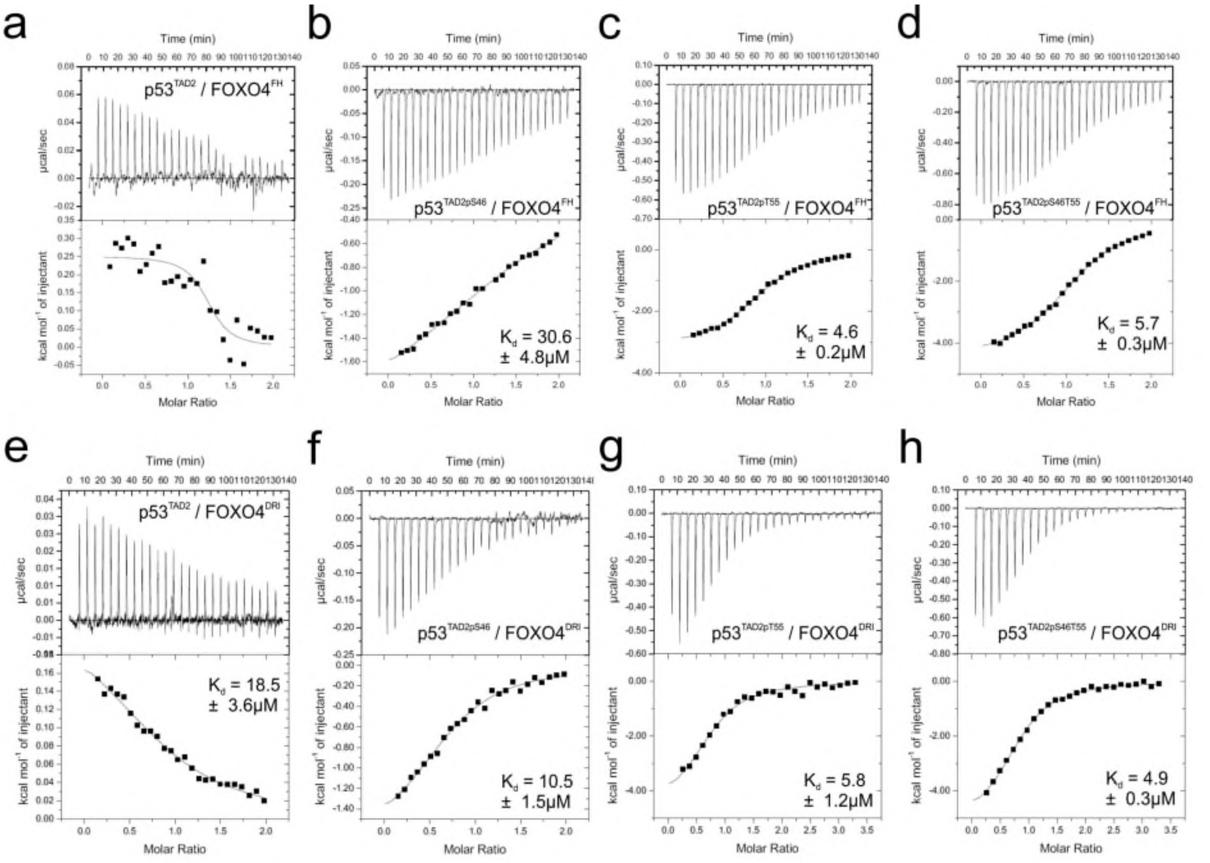

Supplementary Figure 9. Phosphorylation of p53^{TAD2} enhances its affinity for FOXO4^{FH} and FOXO4-DRI: Titration of 500 µM of p53^{TAD2}, p53^{TAD2pS46}, p53^{TADpT55}, and p53^{TAD2pS46T55} to 50 µM FOXO4^{FH} (A-D) or FOXO4-DRI (E-H). The reported errors correspond to the SD of the fit.

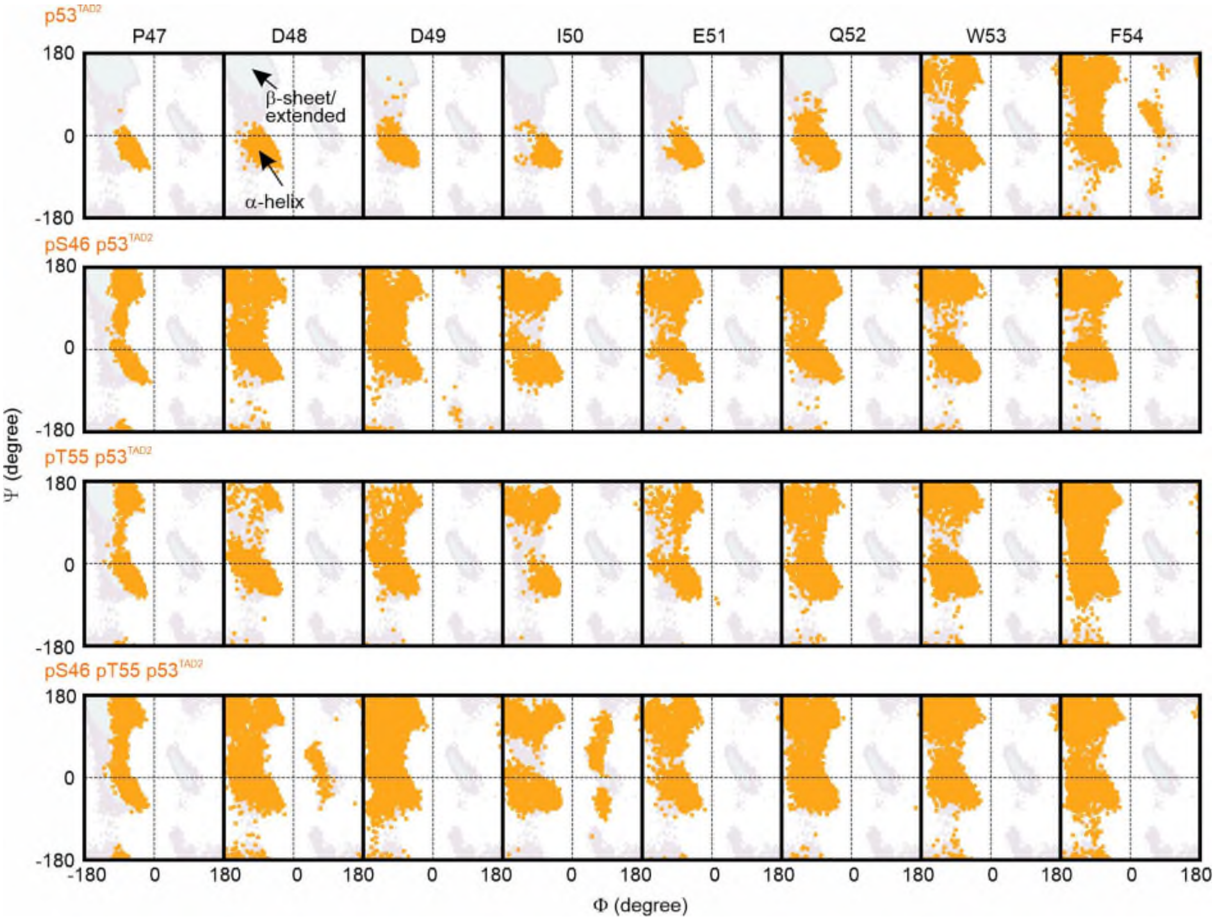

Supplementary Figure 10. Analysis of MD simulations. Ramachandran plot for residues 47-54 in unphosphorylated and phosphorylated p53^{TAD2}, respectively along the different MD simulations.

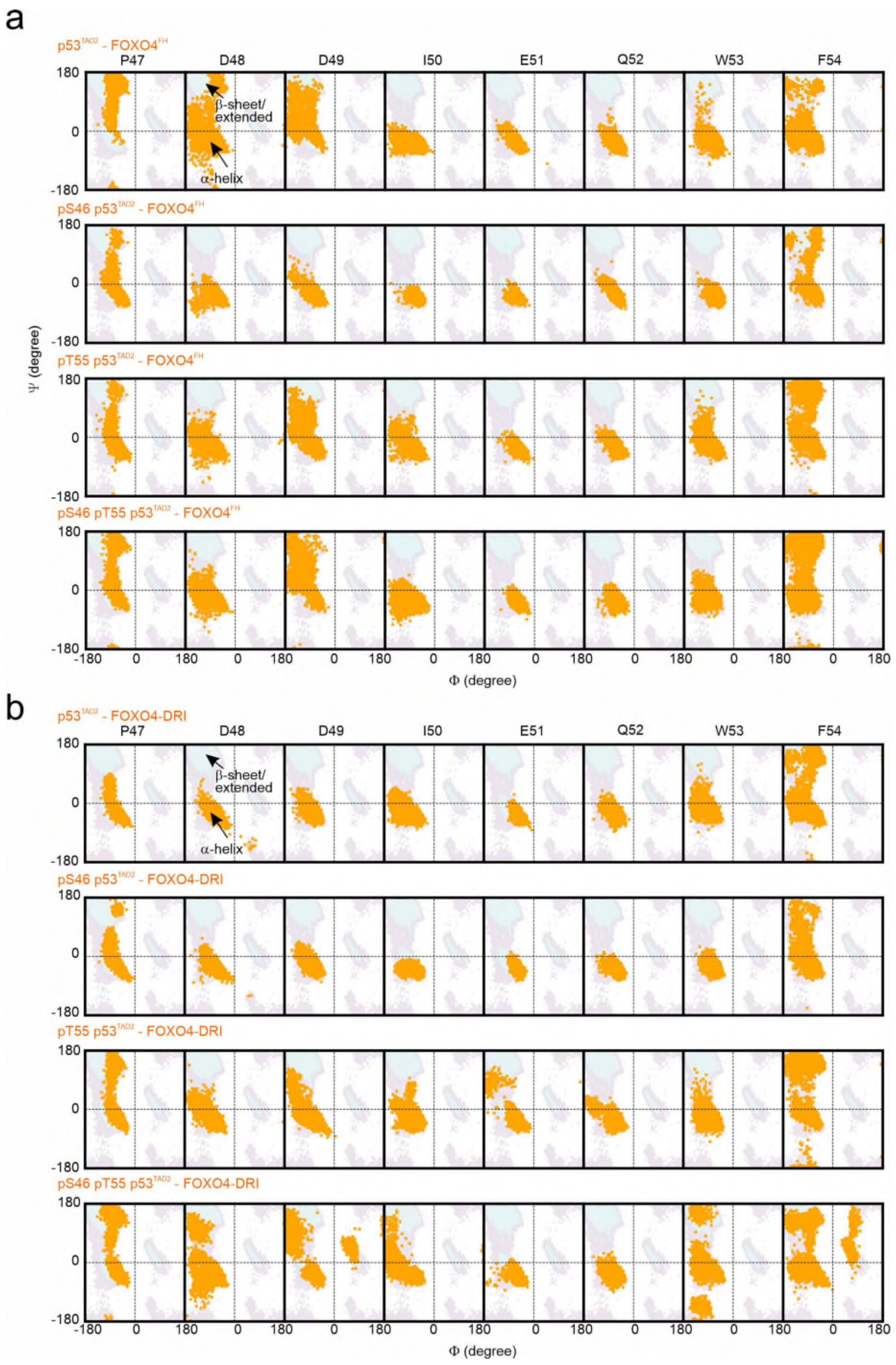
a
p53TAD2 - FOXO4FH
pS46 p53TAD2 - FOXO4FH
pT55 p53TAD2 - FOXO4FH
pS46 pT55 p53TAD2 - FOXO4FH
P47
D48
D49
I50
E51
Q52
W53
F54
β-sheet/ extended
α-helix
Ψ (degree)
Φ (degree)
b
p53TAD2 - FOXO4-DRI
pS46 p53TAD2 - FOXO4-DRI
pT55 p53TAD2 - FOXO4-DRI
pS46 pT55 p53TAD2 - FOXO4-DRI

Supplementary Figure 11. Analysis of MD simulations. Ramachandran plot for residues 47-54 in unphosphorylated phosphorylated p53^{TAD2} along the different MD simulations of p53^{TAD2} in complex with FOXO4^{FH} (a) or FOXO4-DRI (b).

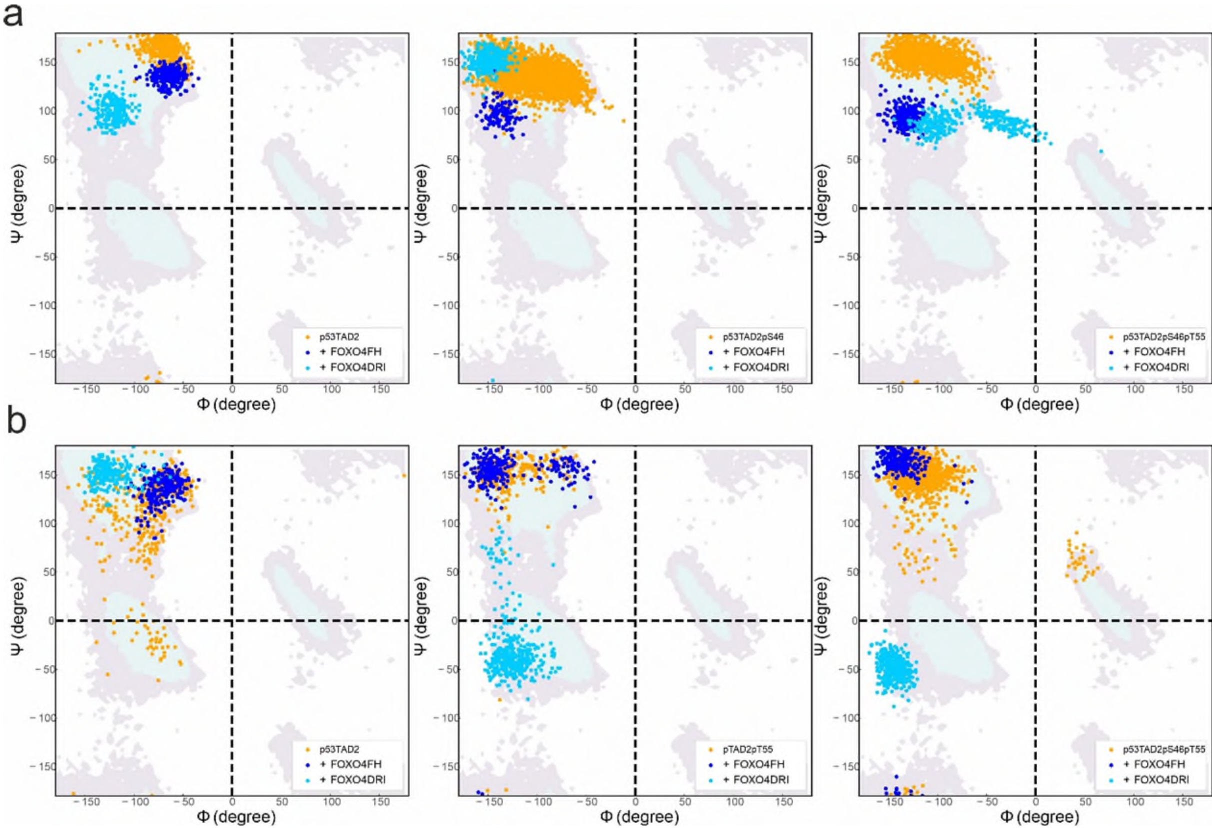

Supplementary Figure 12. Analysis of MD simulations. Ramachandran plot for p53 serine 46 (a) or threonine 55 in the unphosphorylated or phosphorylated states and in the absence of ligand or upon binding to FOXO4^{FH} (dark blue) or FOXO4-DRI (light blue), respectively.

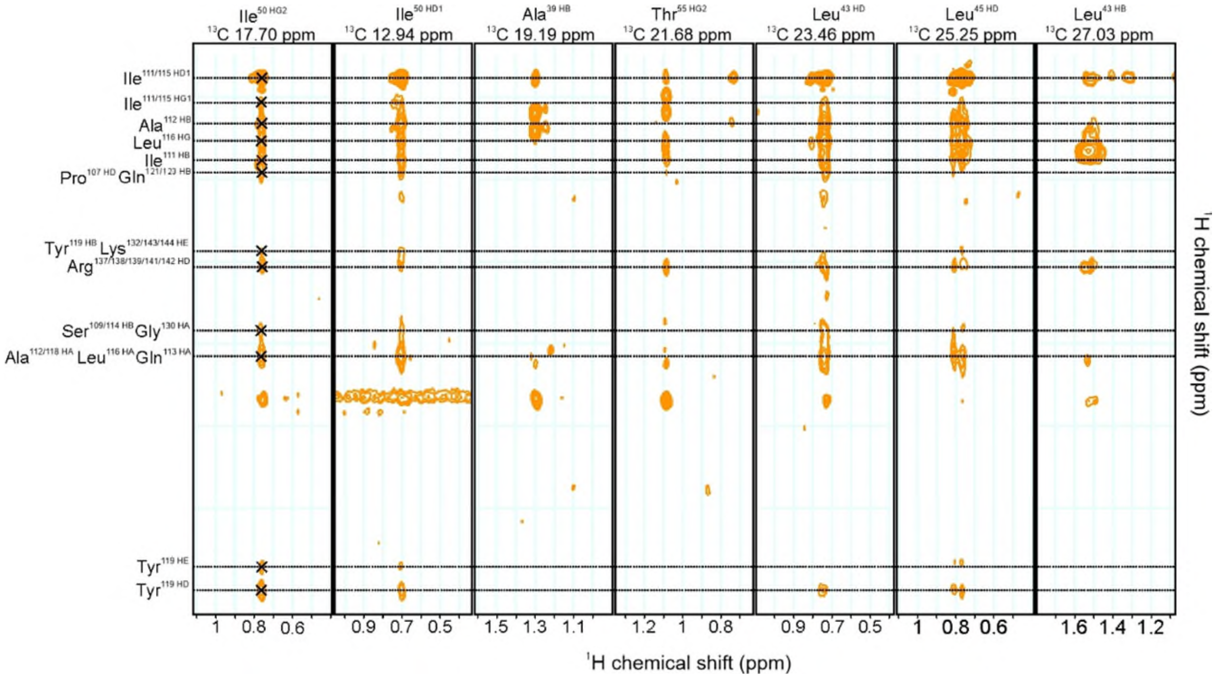

Supplementary Figure 13. Structure determination. Exemplary NOE strips showing intermolecular NOEs between ^{1}H,^{13}C labeled p53^{TAD2} and unlabeled FOXO4^{DRI} extracted from ^{13}C,^{15}N filtered, ^{13}C-edited 3D NOESY-HSQC experiments.

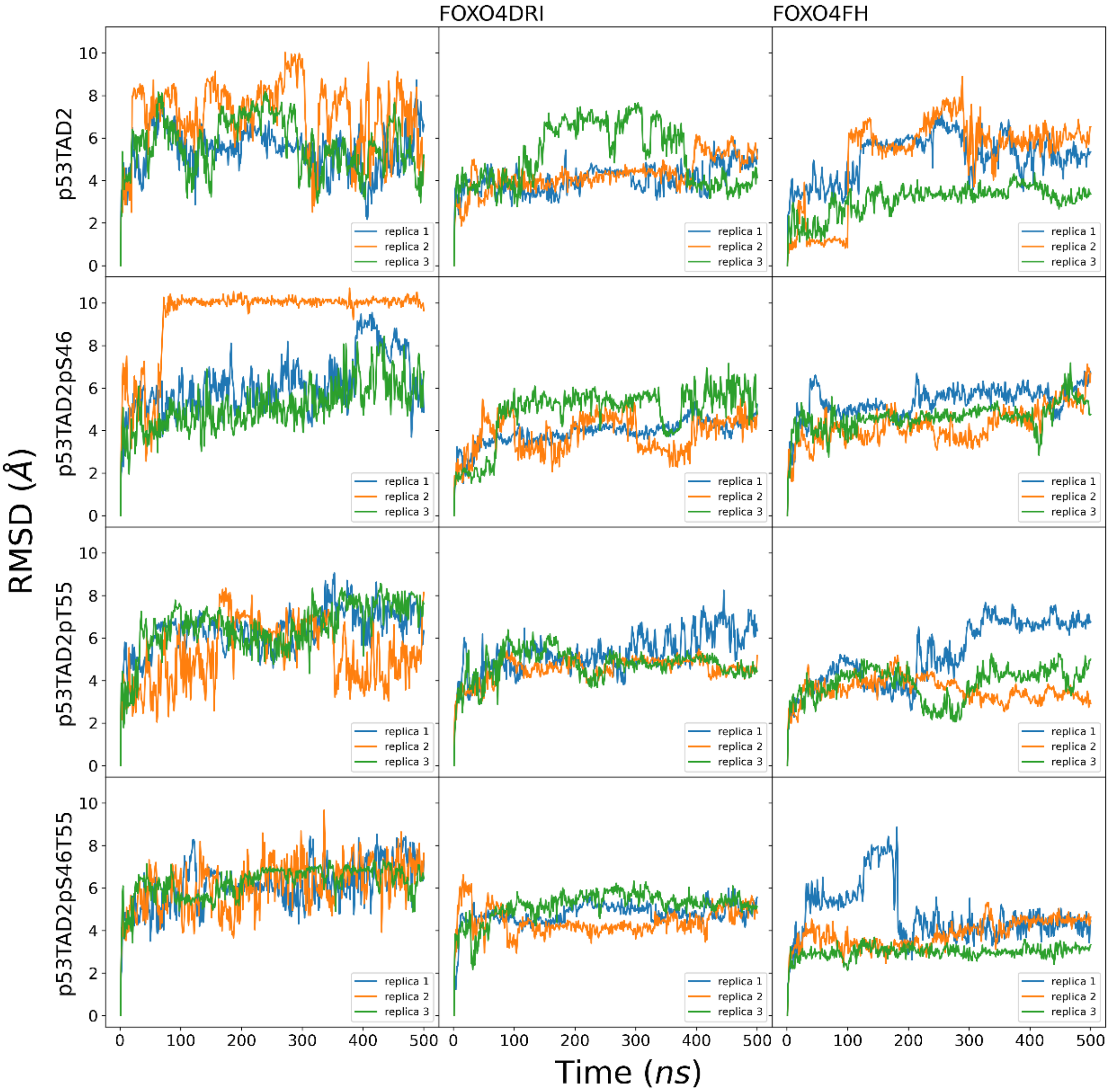

Supplementary Figure 14. Analysis of MD simulations. Evolution of RMSD (Å) for the backbone atoms of p53^{TAD2}, both alone and in complex with the two binding partners, throughout the MD simulations.

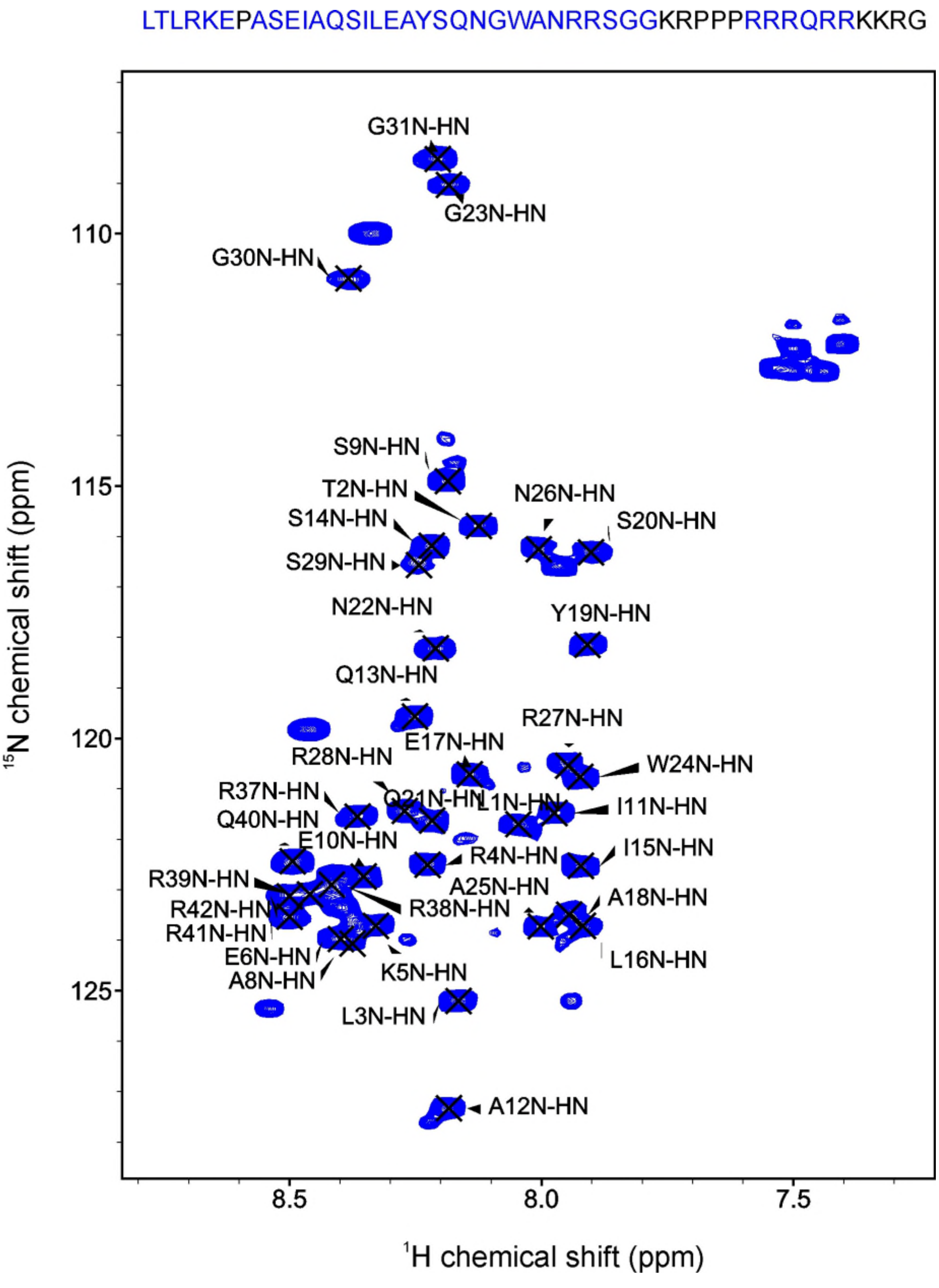

Supplementary Figure 15. ^{1}H,^{15}N HSQC assigned spectrum of ^{15}N-labeled FOXO4^{LRI}. The assigned backbone resonances are indicated in bleu in the corresponding primary amino-acids sequence.

Full blots related to supplementary Fig. 1d

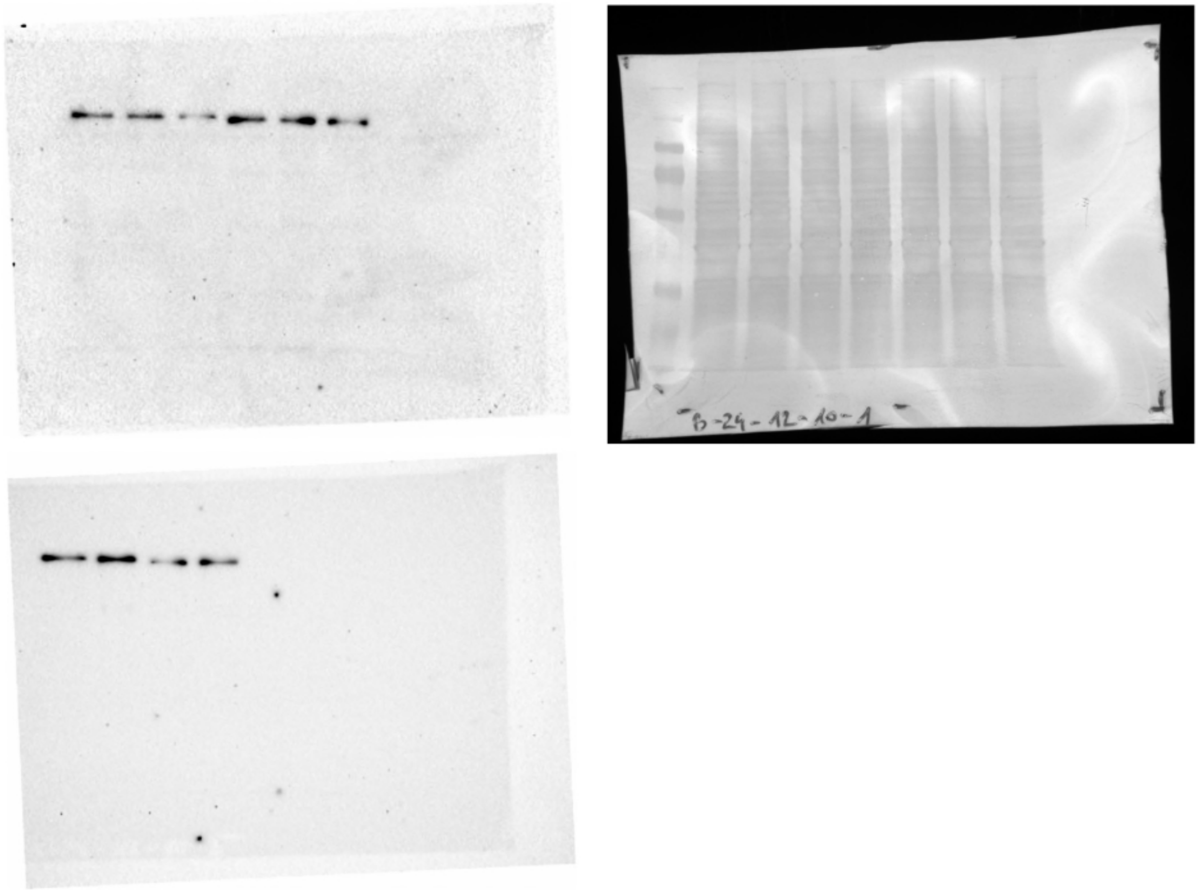

Full blots related to supplementary Fig. 7c

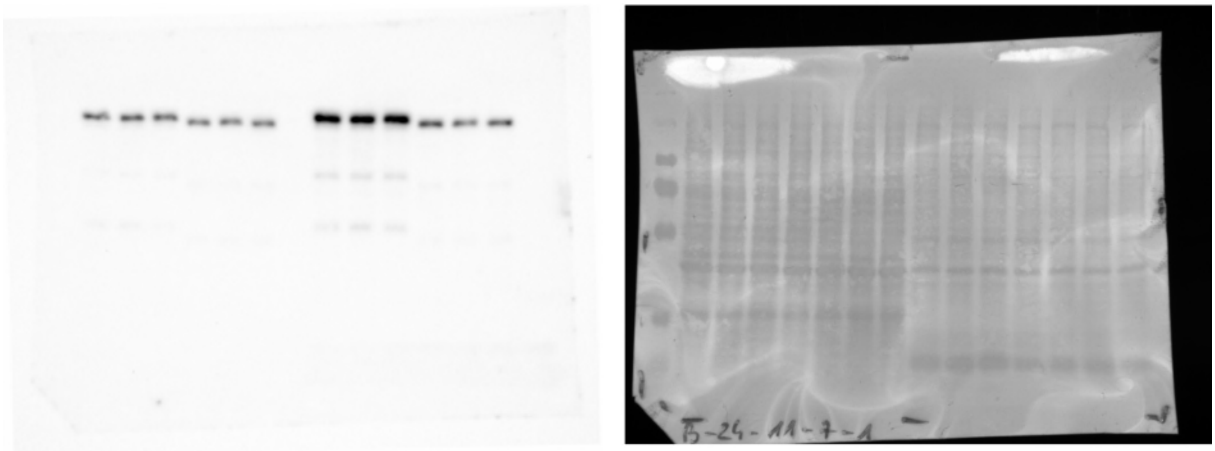

Supplementary Figure 16. Full blots related to supplementary Figure 1d and 7c.

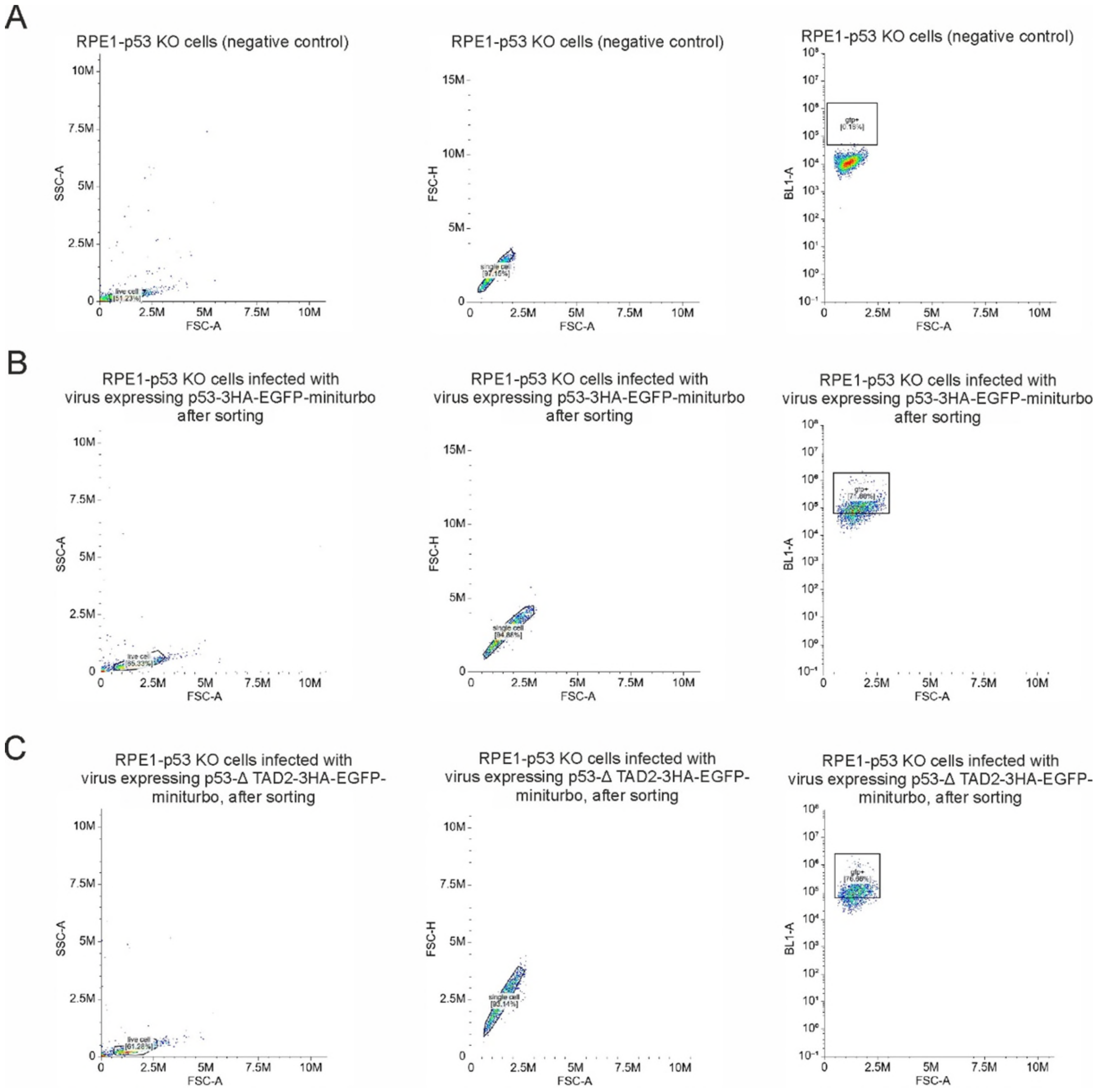

Supplementary Figure 17. FACS data and gating strategy. The gating strategy was: 1) FSC-A (Forward Scatter) vs SSC-A (Side Scatter) to choose live cells, 2) FSC-A (Forward Scatter) vs FSC-H (Forward Scatter) to get single cells from live cell populations, 3) FITC-A vs FSC-A to get GFP-positive cells from that single cell population.

Supplementary Table 1. Details of the simulation setup.

Simulation system	Simulation box dimensions (A^3)	Counter ions	Composition (number of protein molecules)
$p53^{TAD2}$	170356.52	7 x Na^+	1 molecule
$p53^{TAD2}$ pT55	170356.52	8 x Na^+	1 molecule
$p53^{TAD2}$ pS46	170356.52	8 x Na^+	1 molecule
$p53^{TAD2}$ - $FOXO4^{FH}$	431134.80	4 x Cl^-	2 molecules
$p53^{TAD2}$ - FOXO4-DRI	259017.37	3 x Cl^-	2 molecules
$p53^{TAD2}$ pS46 – $FOXO4^{FH}$	431134.80	3 x Cl^-	2 molecules
$p53^{TAD2}$ pS46 – FOXO4-DRI	259017.37	2 x Cl^-	2 molecules
$p53^{TAD2}$ pT55 – $FOXO4^{FH}$	431134.80	2 x Cl^-	2 molecules
$p53^{TAD2}$ pT55 – FOXO4-DRI	259017.37	3 x Cl^-	2 molecules
$p53^{TAD2}$ pS46 pT55 – $FOXO4^{FH}$	431134.80	2 x Cl^-	2 molecules
$p53^{TAD2}$ pS46pT55 – FOXO4-DRI	308389.67	1 x Cl^-	2 molecules

Supplementary Note 1. List of intermolecular NOEs used in the structure calculations of the p53^{TAD2}-FOXO4^{FH} complex. Unambiguously assigned NOEs are colored in blue.

1. assign (residue 50 and name HD* and segid free) ((resi 100 or resi 131 or resi 197 or resi 200) and name H* and segid free) 5.0 3.2 0.0
2. assign (residue 50 and name HD* and segid free) ((resi 105 or resi 107 or resi 108 or resi 136) and name H* and segid free) 5.0 3.2 0.0
3. assign (residue 50 and name HD* and segid free) ((resi 97 or resi 114 or resi 121 or resi 141 or resi 204 or resi 205) and name H* and segid free) 5.0 3.2 0.0
4. assign (residue 50 and name HD* and segid free) ((resi 122 or resi 155 or resi 167 or resi 191 or resi 203) and name H* and segid free) 5.0 3.2 0.0
5. assign ((residue 50 and name HD* and segid free)) (((resi 84 or resi 87 or resi 102 or resi 116 or resi 118 or resi 135 or resi 152 or resi 156 or resi 194 or resi 205 or resi 206) and name H* and segid free)) 5.0 3.2 0.0
6. assign (residue 50 and name HD* and segid free) ((resi 101 or resi 101 or resi 130 or resi 145 or resi 178 or resi 178) and name H* and segid free) 5.0 3.2 0.0
7. assign (residue 50 and name HB and segid free) ((resi 114 or resi 123 or resi 124 or resi 151 or resi 167) and name H* and segid free) 5.0 3.2 0.0
8. assign (residue 50 and name HG* and segid free) ((resi 98 or resi 133 or resi 165 or resi 194 or resi 203 or resi 205 or resi 205) and name H* and segid free) 5.0 3.2 0.0
9. assign ((residue 50 and name HG* and segid free)) (((resi 122 or resi 155 or resi 167 or resi 203) and name H* and segid free)) 5.0 3.2 0.0
10. assign (residue 50 and name HG* and segid free) ((resi 101 or resi 101 or resi 130 or resi 145 or resi 178) and name H* and segid free) 5.0 3.2 0.0
11. assign (residue 50 and name HG* and segid free) ((resi 130 or resi 150 or resi 177 or resi 177) and name H* and segid free) 5.0 3.2 0.0
12. assign (residue 50 and name HG* and segid free) ((resi 97 or resi 98 or resi 101 or resi 114 or resi 120 or resi 133 or resi 156 or resi 161) and name H* and segid free) 5.0 3.2 0.0
13. assign (residue 50 and name HG* and segid free) ((resi 84 or resi 120 or resi 160 or resi 171 or resi 186 or resi 189 or resi 195 or resi 196) and name H* and segid free) 5.0 3.2 0.0
14. assign (residue 55 and name HG* and segid free) ((resi 97:98 or resi 114 or resi 133 or resi 141 or resi 165 or resi 189 or resi 194 or resi 203 or resi 205:206) and name H* and segid free) 5.0 3.2 0.0
15. assign (residue 55 and name HG* and segid free) ((resi 97 or resi 98 or resi 114 or resi 133 or resi 156 or resi 161) and name H* and segid free) 5.0 3.2 0.0
16. assign ((residue 55 and name HG* and segid free)) (((resi 101 or resi 130 or resi 145 or resi 152 or resi 177 or resi 178) and name H* and segid free)) 5.0 3.2 0.0
17. assign (((residue 40 or residue 44) and name HE* and segid free)) (((resi 93 or resi 99 or resi 109 or resi 120 or resi 122 or resi 155 or resi 167 or resi 180 or resi 186 or resi 189 or resi 194 or resi 203) and name H* and segid free)) 5.0 3.2 0.0
18. assign ((residue 40 or residue 44) and name HE* and segid free) ((resi 85 or resi 126 or resi 126 or resi 129 or resi 198) and name H* and segid free) 5.0 3.2 0.0
19. assign ((residue 40 or residue 44) and name HE* and segid free) ((resi 97 or resi 98 or resi 114 or resi 133 or resi 156 or resi 161 or resi 206) and name H* and segid free) 5.0 3.2 0.0
20. assign ((residue 40 or residue 44) and name HE* and segid free) ((resi 88 or resi 126 or resi 142 or resi 163 or resi 167 or resi 172 or resi 172) and name H* and segid free) 5.0 3.2 0.0
21. assign (((residue 40 or residue 44) and name HE* and segid free)) (((resi 85 or resi 89 or resi 91 or resi 96 or resi 99 or resi 104 or resi 105 or resi 159 or resi 168 or resi 175 or resi 182 or resi 191 or resi 197 or resi 200) and name H* and segid free)) 5.0 3.2 0.0
22. assign ((residue 40 or residue 44) and name HE* and segid free) ((resi 101 or resi 101 or resi 101 or resi 145 or resi 178 or resi 178) and name H* and segid free) 5.0 3.2 0.0
23. assign ((residue 40 or residue 44) and name HE* and segid free) ((resi 130 or resi 177 or resi 178 or resi 178) and name H* and segid free) 5.0 3.2 0.0
24. assign ((residue 40 or residue 44) and name HE* and segid free) ((resi 99 or resi 101 or resi 103 or resi 104 or resi 126 or resi 130 or resi 169 or resi 178) and name H* and segid free) 5.0 3.2 0.0
25. assign ((residue 40 or residue 44) and name HE* and segid free) ((resi 113 or resi 116 or resi 130 or resi 117 or resi 178 or resi 178) and name H* and segid free) 5.0 3.2 0.0
26. assign ((residue 40 or residue 44) and name HE* and segid free) ((resi 99 or resi 112 or resi 126 or resi 133 or resi 141 or resi 145 or resi 152 or resi 155 or resi 157 or resi 181) and name H* and segid free) 5.0 3.2 0.0
27. assign ((residue 40 or residue 44) and name HE* and segid free) ((resi 91 or resi 102 or resi 118 or resi 155 or resi 191) and name H* and segid free) 5.0 3.2 0.0
28. assign ((residue 40 or residue 44) and name HE* and segid free) ((resi 101 or resi 120 or resi 137) and name H* and segid free) 5.0 3.2 0.0
29. assign ((residue 40 or residue 44) and name HE* and segid free) ((resi 110) and name H* and segid free) 5.0 3.2 0.0
30. assign (((residue 43 or residue 45) and name HD* and segid free)) (((resi 84 or resi 87 or resi 107 or resi 120 or resi 120 or resi 141 or resi 148 or resi 160 or resi 171 or resi 186 or resi 195 or resi 196) and name H* and segid free)) 5.0 3.2 0.0
31. assign ((residue 43 or residue 45) and name HD* and segid free) ((resi 97 or resi 114 or resi 121 or resi 133 or resi 141 or resi 194 or resi 204 or resi 204 or resi 205 or resi 206) and name H* and segid free) 5.0 3.2 0.0

32. assign ((residue 43 or residue 45) and name HD* and segid free) ((resi 97 or resi 98 or resi 114 or resi 133 or resi 156 or resi 161 or resi 206) and name H* and segid free) 5.0 3.2 0.0
33. assign (((residue 43 or residue 45) and name HD* and segid free)) (((resi 83 or resi 94 or resi 95 or resi 111 or resi 115 or resi 125 or resi 130 or resi 137 or resi 142 or resi 147 or resi 148 or resi 149 or resi 159 or resi 162 or resi 184 or resi 185 or resi 188) and name H* and segid free)) 5.0 3.2 0.0
34. assign (((residue 43 or residue 45) and name HD* and segid free)) (((resi 88 or resi 93 or resi 101 or resi 102 or resi 120 or resi 157 or resi 160 or resi 166 or resi 171 or resi 176 or resi 183 or resi 194 or resi 195 or resi 196 or resi 203 or resi 204) and name H* and segid free)) 5.0 3.2 0.0
35. assign (((residue 43 or residue 45) and name HD* and segid free)) (((resi 89 or resi 91 or resi 96 or resi 99 or resi 105 or resi 157 or resi 168 or resi 175 or resi 182 or resi 191 or resi 197 or resi 206) and name H* and segid free)) 5.0 3.2 0.0
36. assign ((residue 43 or residue 45) and name HD* and segid free) ((resi 130 or resi 177 or resi 178 or resi 178) and name H* and segid free) 5.0 3.2 0.0
37. assign (((residue 43 or residue 45) and name HD* and segid free)) (((resi 85 or resi 104 or resi 109 or resi 113 or resi 115 or resi 118 or resi 132 or resi 137 or resi 170 or resi 179 or resi 182 or resi 198) and name H* and segid free)) 5.0 3.2 0.0
38. assign (((residue 43 or residue 45) and name HD* and segid free)) (((resi 91 or resi 115 or resi 118 or resi 129 or resi 136 or resi 138 or resi 141 or resi 157 or resi 179 or resi 182 or resi 183 or resi 191) and name H* and segid free)) 5.0 3.2 0.0
39. assign (((residue 43 or residue 45) and name HD* and segid free)) (((resi 89 or resi 106 or resi 120 or resi 126 or resi 142 or resi 144 or resi 153 or resi 157 or resi 160 or resi 166 or resi 172 or resi 183) and name H* and segid free)) 5.0 3.2 0.0
40. assign ((resi 160 and name HD*) and segid free) ((resi 53 and name HE*) and segid free) 5.0 3.2 0.0
41. assign ((resi 109 and name HD*) and segid free) ((resi 53 and name HD*) and segid free) 5.0 3.2 0.0
42. assign (((resi 85 or resi 131 or resi 179 or resi 198) and name HE* and segid free)) (((resi 43 and name HD*) or (resi 45 and name HD*) or (resi 50 and name HG*) and segid free)) 5.0 3.2 0.0
43. assign (((resi 85 and name HE*) or (resi 131 and name HE*) or (resi 179 and name HE*) or (resi 198 and name HE*) and segid free)) (((resi 43 and name HB*) and segid free)) 5.0 3.2 0.0
44. assign (((resi 88 or resi 89 or resi 132 or resi 134) and name HG* and segid free)) (((resi 52 and name HA) or (resi 55 and name HB) and segid free)) 5.0 3.2 0.0
45. assign (((resi 88 or resi 89 or resi 132 or resi 134) and name HG* and segid free)) (((resi 51 and name HA) or (resi 52 and name HA) or (resi 55 and name HB) and segid free)) 5.0 3.2 0.0
46. assign (((resi 160 and name HD*) and segid free)) (((resi 38 or resi 40 or resi 40 or resi 44 or resi 44 or resi 47) and name H* and segid free)) 5.0 3.2 0.0
47. assign (((resi 204 or resi 205) and name HD* and segid free)) (((resi 43 and name HB*) or (resi 43 and name HG*) or (resi 45 and name HB*) or (resi 45 and name HG*) and segid free)) 5.0 3.2 0.0
48. assign (((resi 204 and name HD*) or (resi 205 and name HD*) and segid free))
(((resi 40 or resi 44 or resi 45) and name HA and segid free)) 5.0 3.2 0.0

Supplementary Note 2. List of intermolecular NOEs used in the structure calculations of the p53[TAD2]-FOXO4-DRI complex. Unambiguously assigned NOEs are colored in blue.

1. assign (resi 102 and name HG* and segid fdri) ((resi 43 or resi 45 or resi 50) and name HD* and segid free) 5.0 3.2 0.0 !685
2. assign (resi 102 and name HG* and segid fdri) ((resi 51:52) and name HB* and segid free) 5.0 3.2 0.0 !686
3. assign (resi 102 and name HG* and segid fdri) ((resi 41:42 or resi 48:49 or resi 56) and name HB* and segid free) 5.0 3.2 0.0 !687 !any Asp HB
4. assign (resi 102 and name HG* and segid fdri) ((resi 38:39 or resi 55:57) and name HB* and segid free) 5.0 3.2 0.0 !688
5. assign ((resi 111 or resi 115) and name HD* and segid fdri) ((resi 43 or resi 45 or resi 50) and name HD* and segid free) 5.0 3.2 0.0 !675
6. assign ((resi 111 or resi 115) and name HD* and segid fdri) ((resi 50) and name HG* and segid free) 5.0 3.2 0.0 !766
7. assign ((resi 111 or resi 115) and name HD* and segid fdri) ((resi 43 or resi 45) and name HG* and segid free) 5.0 3.2 0.0 !767
8. assign ((resi 111 or resi 115) and name HD* and segid fdri) ((resi 47 or resi 51:52 or resi 56) and name HB* and segid free) 5.0 3.2 0.0 !676
9. assign ((resi 111 or resi 115) and name HD* and segid fdri) ((resi 51:52) and name HG* and segid free) 5.0 3.2 0.0 !677
10. assign ((resi 111 or resi 115) and name HD* and segid fdri) ((resi 41:42 or resi 48:49 or resi 56) and name HB* and segid free) 5.0 3.2 0.0 !768 !any Asp HB !weak peak
11. assign ((resi 111 or resi 115) and name HD* and segid fdri) ((resi 53:54) and name HB* and segid free) 5.0 3.2 0.0 !769
12. assign ((resi 111 or resi 115) and name HD* and segid fdri) ((resi 50) and name HA and segid free) 5.0 3.2 0.0 !770
13. assign ((resi 111 or resi 115) and name HD* and segid fdri) ((resi 40 or resi 45 or resi 47) and name HA and segid free) 5.0 3.2 0.0 !680
14. assign ((resi 111 or resi 115) and name HD* and segid fdri) ((resi 53:54) and (name HE* or name HD* or name HH*) and segid free) 5.0 3.2 0.0 !771
15. assign ((resi 115) and name HG2* and segid fdri) ((resi 43 or resi 45 or resi 50) and name HD* and segid free) 5.0 3.2 0.0 !682
16. assign ((resi 115) and name HG2* and segid fdri) ((resi 50 or resi 55) and name HG* and segid free) 5.0 3.2 0.0 !772
17. assign ((resi 115) and name HG2* and segid fdri) ((resi 50) and name HG* and segid free) 5.0 3.2 0.0
18. assign ((resi 115) and name HG2* and segid fdri) ((resi 43 or resi 45) and name HG* and segid free) 5.0 3.2 0.0
19. assign ((resi 115) and name HG2* and segid fdri) ((resi 43 or resi 45) and name HB* and segid free) 5.0 3.2 0.0
20. assign ((resi 115) and name HG2* and segid fdri) ((resi 50 or resi 52) and name HG* and segid free) 5.0 3.2 0.0 !774
21. assign ((resi 115) and name HG2* and segid fdri) ((resi 54) and name HB* and segid free) 5.0 3.2 0.0 !776 !very weak peak
22. assign ((resi 115) and name HG2* and segid fdri) ((resi 50:51) and name HA and segid free) 5.0 3.2 0.0 !684
23. assign ((resi 111) and name HG2* and segid fdri) ((resi 45 or resi 50) and (name HB* or name HG*) and segid free) 5.0 3.2 0.0 !772
24. assign ((resi 111) and name HG2* and segid fdri) ((resi 47 or resi 51:52 or resi 56) and name HB* and segid free) 5.0 3.2 0.0 !683
25. assign ((resi 111) and name HG2* and segid fdri) ((resi 53) and name HB* and segid free) 5.0 3.2 0.0 !777 !very weak peak
26. assign ((resi 111 or resi 115) and name HG2* and segid fdri) ((resi 43 or resi 45) and (name HB* or name HG*) and segid free) 5.0 3.2 0.0 !773
27. assign ((resi 111 or resi 115) and name HG2* and segid fdri) ((resi 53:54) and (name HE* or name HD* or name HH*) and segid free) 5.0 3.2 0.0 !771
28. assign (resi 112 and name HB* and segid fdri) (resi 50 and name HD* and segid free) 5.0 3.2 0.0
29. assign (resi 112 and name HB* and segid fdri) ((resi 43 or resi 45) and name HD* and segid free) 5.0 3.2 0.0
30. assign (resi 112 and name HB* and segid fdri) (resi 50 and name HG2* and segid free) 5.0 3.2 0.0
31. assign (resi 112 and name HB* and segid fdri) (resi 43 and name HD* and segid free) 5.0 3.2 0.0
32. assign (resi 112 and name HB* and segid fdri) ((resi 50 or resi 55) and name HG* and segid free) 5.0 3.2 0.0
33. assign (resi 112 and name HB* and segid fdri) ((resi 43 or resi 45) and name HB* and segid free) 5.0 3.2 0.0
34. assign (resi 112 and name HB* and segid fdri) ((resi 47 or resi 51:52 or resi 56) and name HB* and segid free) 5.0 3.2 0.0 !672
35. assign (resi 112 and name HB* and segid fdri) ((resi 47 or resi 51:52 or resi 56) and (name HB* or name HG*) and segid free) 5.0 3.2 0.0 !673

36. assign (resi 112 and name HB* and segid fdri) ((resi 40:42 or resi 44 or resi 48:49 or resi 56) and name HB* and segid free) 5.0 3.2 0.0 !778 !any Asp /MET HB
37. assign (resi 112 and name HB* and segid fdri) ((resi 41:42 or resi 48:49 or resi 56) and name HB* and segid free) 5.0 3.2 0.0 !779 !any Asp HB !weak peak
38. assign (resi 112 and name HB* and segid fdri) ((resi 53:54) and name HB* and segid free) 5.0 3.2 0.0
39. assign (resi 112 and name HB* and segid fdri) (resi 47 and name HD* and segid free) 5.0 3.2 0.0
40. assign (resi 112 and name HB* and segid fdri) ((resi 50:51) and name HA and segid free) 5.0 3.2 0.0 !780
41. assign (resi 112 and name HB* and segid fdri) ((resi 38:39 or resi 47 or resi 55:57) and name HA and segid free) 5.0 3.2 0.0 !674
42. assign (resi 112 and name HB* and segid fdri) ((resi 48:49 or resi 53) and name HA and segid free) 5.0 3.2 0.0 !781
43. assign (resi 112 and name HB* and segid fdri) ((resi 53) and name HE* and segid free) 5.0 3.2 0.0
44. assign (resi 112 and name HB* and segid fdri) ((resi 54) and (name HE* or name HD* or name HH*) and segid free) 5.0 3.2 0.0 !782
45. assign (resi 116 and name HD* and segid fdri) ((resi 43 or resi 45 or resi 50) and name HD* and segid free) 5.0 3.2 0.0 !787
46. assign (resi 116 and name HD* and segid fdri) ((resi 43 or resi 45) and (name HB* or name HG*) and segid free) 5.0 3.2 0.0 !788
47. assign (resi 116 and name HD* and segid fdri) ((resi 50:52 or resi 56) and name HB* and segid free) 5.0 3.2 0.0 !789
48. assign (resi 116 and name HD* and segid fdri) ((resi 38 or resi 40 or resi 44 or resi 47) and name HB* and segid free) 5.0 3.2 0.0 !790
49. assign (resi 116 and name HD* and segid fdri) ((resi 37 or resi 40 or resi 45) and name HA and segid free) 5.0 3.2 0.0 !791
50. assign (resi 116 and name HD* and segid fdri) ((resi 54) and (name HE* or name HD* or name HH*) and segid free) 5.0 3.2 0.0 !782
51. assign (resi 116 and name HB* and segid fdri) (resi 50 and name HD* and segid free) 5.0 3.2 0.0
52. assign (resi 118 and name HB* and segid fdri) ((resi 43 or resi 45 or resi 50) and name HD* and segid free) 5.0 3.2 0.0 !783
53. assign (resi 118 and name HB* and segid fdri) (resi 51 and name HA and segid free) 5.0 3.2 0.0
54. assign (resi 118 and name HB* and segid fdri) ((resi 42 or resi 48) and name HA and segid free) 5.0 3.2 0.0 !671 !HA probably more ambig
55. assign (resi 118 and name HB* and segid fdri) ((resi 53) and (name HD* or name HZ*) and segid free) 5.0 3.2 0.0
56. assign (resi 125 and name HB* and segid fdri) ((resi 38 or resi 40 or resi 44 or resi 52 or resi 56) and name HB* and segid free) 5.0 3.2 0.0 !785 !broad peak
57. assign (resi 125 and name HB* and segid fdri) ((resi 53:54) and (name HE* or name HD* or name HH*) and segid free) 5.0 3.2 0.0 !771
58. assign (resi 39 and name HB* and segid free) ((resi 115 or resi 116 or resi 101 or resi 103 or resi 111) and name HD* and segid fdri) 5.0 3.2 0.0
59. assign ((resi 40 or resi 44) and name HE* and segid free) ((resi 111 or resi 115 or resi 116) and (name HD* or name HG*) and segid fdri) 5.0 3.2 0.0 !548
60. assign ((resi 40 or resi 44) and name HE* and segid free) (resi 119 and name HE* and segid fdri) 5.0 3.2 0.0
61. assign ((resi 40 or resi 44) and name HE* and segid free) (resi 119 and name HD* and segid fdri) 5.0 3.2 0.0
62. assign ((resi 40 or resi 44) and name HE* and segid free) ((resi 124) and (name HE* or name HZ*) and segid fdri) 5.0 3.2 0.0
63. assign ((resi 43 or resi 45) and name HD* and segid free) ((resi 111 or resi 115 or resi 116) and (name HD*) and segid fdri) 5.0 3.2 0.0
64. assign ((resi 43 or resi 45) and name HD* and segid free) ((resi 118 or resi 125) and (name HB*) and segid fdri) 5.0 3.2 0.0
65. assign ((resi 43 or resi 45) and name HD* and segid free) (resi 112 and (name HB*) and segid fdri) 5.0 3.2 0.0
66. assign ((resi 43 or resi 45) and name HD* and segid free) ((resi 106:107 or resi 110 or resi 117 or resi 134:136) and (name HB* or name HG*) and segid fdri) 5.0 3.2 0.0 !713
67. assign ((resi 43 or resi 45) and name HD* and segid free) ((resi 122 or resi 126) and (name HB*) and segid fdri) 5.0 3.2 0.0
68. assign ((resi 43 or resi 45) and name HD* and segid free) ((resi 107 or resi 134:136) and (name HB*) and segid fdri) 5.0 3.2 0.0 !714
69. assign ((resi 43 or resi 45) and name HD* and segid free) ((resi 109 or resi 114 or resi 120 or resi 123 or resi 129:131) and (name HA* or name HB*) and segid fdri) 5.0 3.2 0.0 !715
70. assign ((resi 43 or resi 45) and name HD* and segid free) ((resi 119 or resi 124) and (name HD* or name HZ*) and segid fdri) 5.0 3.2 0.0
71. assign ((resi 43 or resi 45) and name HG* and segid free) ((resi 111 or resi 115 or resi 116) and (name HD*) and segid fdri) 5.0 3.2 0.0
72. assign (resi 43 and name HD1* and segid free) ((resi 111 or resi 115 or resi 116) and (name HD*) and segid fdri) 5.0 3.2 0.0
73. assign (resi 43 and name HD1* and segid free) ((resi 112) and name HB* and segid fdri) 5.0 3.2 0.0

74. assign (resi 43 and name HD1* and segid free) ((resi 119) and name HD* and segid fdri) 5.0 3.2 0.0
75. assign (resi 43 and name HD1* and segid free) ((resi 104 or resi 106:107 or resi 110 or resi 113 or resi 117 or resi 121 or resi 134:136 or resi 140) and (name HB* or name HG*) and segid fdri) 5.0 3.2 0.0 !723 Pro HG Gln/Glu HB*
76. assign (resi 43 and name HD1* and segid free) ((resi 119) and name HE* and segid fdri) 5.0 3.2 0.0
77. assign (resi 45 and name HD1* and segid free) ((resi 111 or resi 115 or resi 116) and (name HD*) and segid fdri) 5.0 3.2 0.0
78. assign (resi 45 and name HD1* and segid free) ((resi 125) and (name HB*) and segid fdri) 5.0 3.2 0.0
79. assign (resi 45 and name HD1* and segid free) ((resi 125) and name HB* and segid fdri) 5.0 3.2 0.0
80. assign (resi 45 and name HD1* and segid free) ((resi 112) and name HB* and segid fdri) 5.0 3.2 0.0
81. assign (resi 45 and name HD1* and segid free) ((resi 109 or resi 114 or resi 120 or resi 123 or resi 129:131 or resi 146) and (name HA* or name HB*) and segid fdri) 5.0 3.2 0.0 !727
82. assign (resi 45 and name HD1* and segid free) ((resi 119) and name HD* and segid fdri) 5.0 3.2 0.0
83. assign (resi 45 and name HD1* and segid free) ((resi 119) and name HE* and segid fdri) 5.0 3.2 0.0
84. assign (resi 47 and name HB* and segid free) ((resi 111 or resi 115 or resi 116) and (name HD* or name HG2*) and segid fdri) 5.0 3.2 0.0
85. assign (resi 47 and name HG1* and segid free) ((resi 111 or resi 115 or resi 116) and (name HD* or name HG2*) and segid fdri) 5.0 3.2 0.0
86. assign (resi 47 and name HG2* and segid free) ((resi 111 or resi 115 or resi 116) and (name HD* or name HG2*) and segid fdri) 5.0 3.2 0.0
87. assign (resi 47 and name HG* and segid free) ((resi 104 or resi 106:107 or resi 110 or resi 113 or resi 117 or resi 121 or resi 134:136 or resi 140) and (name HB* or name HG*) and segid fdri) 5.0 3.2 0.0 !701 Pro HN Gln/Glu HG*
88. assign (resi 47 and name HG* and segid free) ((resi 107 or resi 134:136) and name HD* and segid fdri) 5.0 3.2 0.0 !743
89. assign (resi 47 and name HG* and segid free) ((resi 124) and name HD1* and segid fdri) 5.0 3.2 0.0 !very weak peak
90. assign (resi 47 and name HG* and segid free) ((resi 124) and name HZ2* and segid fdri) 5.0 3.2 0.0 !very weak peak
91. assign (resi 50 and name HD* and segid free) ((resi 111 or resi 115) and name HD* and segid fdri) 5.0 3.2 0.0
92. assign (resi 50 and name HD* and segid free) ((resi 111 or resi 115) and name HG* and segid fdri) 5.0 3.2 0.0
93. assign (resi 50 and name HD* and segid free) ((resi 118 or resi 125) and name HB* and segid fdri) 5.0 3.2 0.0
94. assign (resi 50 and name HD* and segid free) (resi 112 and name HB* and segid fdri) 5.0 3.2 0.0
95. assign (resi 50 and name HD* and segid free) ((resi 106:107 or resi 110 or resi 117 or resi 134:136) and (name HB* or name HG*) and segid fdri) 5.0 3.2 0.0 !701
96. assign (resi 50 and name HD* and segid free) ((resi 107 or resi 120 or resi 134:136) and (name HD*) and segid fdri) 5.0 3.2 0.0 !703
97. assign (resi 50 and name HD* and segid free) ((resi 109 or resi 114 or resi 120 or resi 123 or resi 129:131) and (name HA* or name HB*) and segid fdri) 5.0 3.2 0.0 !704
98. assign (resi 50 and name HD* and segid free) (resi 119 and name HE* and segid fdri) 5.0 3.2 0.0
99. assign (resi 50 and name HD* and segid free) (resi 119 and name HD* and segid fdri) 5.0 3.2 0.0
100. assign (resi 50 and name HG* and segid free) ((resi 111 or resi 115) and name HD* and segid fdri) 5.0 3.2 0.0
101. assign (resi 50 and name HG* and segid free) ((resi 111 or resi 115) and name HG* and segid fdri) 5.0 3.2 0.0
102. assign (resi 50 and name HG* and segid free) (resi 112 and name HB* and segid fdri) 5.0 3.2 0.0
103. assign (resi 50 and name HG* and segid free) (resi 116 and name HG* and segid fdri) 5.0 3.2 0.0
104. assign (resi 50 and name HG* and segid free) (resi 111 and name HB* and segid fdri) 5.0 3.2 0.0
105. assign (resi 50 and name HG* and segid free) (resi 119 and name HE* and segid fdri) 5.0 3.2 0.0
106. assign (resi 50 and name HG* and segid free) (resi 119 and name HD* and segid fdri) 5.0 3.2 0.0
107. assign (resi 50 and name HB and segid free) ((resi 111 or resi 115 or resi 116) and name HD* and segid fdri) 5.0 3.2 0.0
108. assign (resi 52 and name HG* and segid free) ((resi 111 or resi 115 or resi 116) and (name HD1* or name HG2*) and segid fdri) 5.0 3.2 0.0 !745
109. assign (resi 53 and name HB* and segid free) ((resi 111 or resi 115 or resi 116) and (name HD1* or name HG2*) and segid fdri) 5.0 3.2 0.0 !764
110. assign (resi 53 and name HB* and segid free) ((resi 117 or resi 121 or resi 107 or resi 134:136 or resi 111 or resi 115) and name HB* and segid fdri) 5.0 3.2 0.0 !752
111. assign (resi 54 and name HB1 and segid free) ((resi 111 or resi 115 or resi 116) and (name HD1* or name HG2*) and segid fdri) 5.0 3.2 0.0 !761
112. assign (resi 54 and name HB2 and segid free) ((resi 111 or resi 115 or resi 116) and (name HD1* or name HG2*) and segid fdri) 5.0 3.2 0.0 !760
113. assign (resi 55 and name HG* and segid free) ((resi 111 or resi 115:116) and name HD* and segid fdri) 5.0 3.2 0.0
114. assign (resi 55 and name HG* and segid free) ((resi 117:118 or resi 125) and name HA and segid fdri) 5.0 3.2 0.0

115. assign ((resi 51 or resi 52 or resi 56) and name HB* and segid free) ((resi 111 or resi 115 or resi 116) and (name HD1* or name HG2*) and segid fdri) 5.0 3.2 0.0 !754
116. assign ((resi 51 or resi 52 or resi 56) and name HB* and segid free) ((resi 107 or resi 134:136) and name HD* and segid fdri) 5.0 3.2 0.0 !743
117. assign ((resi 51 or resi 52 or resi 56) and name HB* and segid free) ((resi 111 or resi 115) and (name HG1*) and segid fdri) 5.0 3.2 0.0 !792
118. assign ((resi 51 or resi 52 or resi 56) and name HB* and segid free) ((resi 111 or resi 115) and (name HA) and segid fdri) 5.0 3.2 0.0 !793
119. assign ((resi 51 or resi 52 or resi 56) and name HB* and segid free) ((resi 106 or resi 124 or resi 134:136) and (name HA) and segid fdri) 5.0 3.2 0.0 !794
