# Supplementary material for: The disordered p53 transactivation domain is the target of FOXO4 and the senolytic compound FOXO4-DRI
Source: Nat Commun. 2025 Jul 1;16:5672. doi: 10.1038/s41467-025-60844-9 (PMC12216184; doi:10.1038/s41467-025-60844-9)
Supplement: Supplementary file 2 — Reporting Summary [file 41467_2025_60844_MOESM2_ESM.pdf]

nature portfolio

Corresponding author(s): Tobias Madl

Last updated by author(s): Jun 2, 2025

Reporting Summary

Nature Portfolio wishes to improve the reproducibility of the work that we publish. This form provides structure for consistency and transparency in reporting. For further information on Nature Portfolio policies, see our Editorial Policies and the Editorial Policy Checklist.

Statistics

For all statistical analyses, confirm that the following items are present in the figure legend, table legend, main text, or Methods section.

n/a	Confirmed	
☐	☒	The exact sample size (n) for each experimental group/condition, given as a discrete number and unit of measurement
☒	☐	A statement on whether measurements were taken from distinct samples or whether the same sample was measured repeatedly
☒	☐	The statistical test(s) used AND whether they are one- or two-sided<br>*Only common tests should be described solely by name; describe more complex techniques in the Methods section.*
☒	☐	A description of all covariates tested
☒	☐	A description of any assumptions or corrections, such as tests of normality and adjustment for multiple comparisons
☐	☒	A full description of the statistical parameters including central tendency (e.g. means) or other basic estimates (e.g. regression coefficient) AND variation (e.g. standard deviation) or associated estimates of uncertainty (e.g. confidence intervals)
☒	☐	For null hypothesis testing, the test statistic (e.g. F, t, r) with confidence intervals, effect sizes, degrees of freedom and P value noted<br>*Give P values as exact values whenever suitable.*
☒	☐	For Bayesian analysis, information on the choice of priors and Markov chain Monte Carlo settings
☒	☐	For hierarchical and complex designs, identification of the appropriate level for tests and full reporting of outcomes
☒	☐	Estimates of effect sizes (e.g. Cohen's d, Pearson's r), indicating how they were calculated

Our web collection on statistics for biologists contains articles on many of the points above.

Software and code

Policy information about availability of computer code

Data collection	Topspin 4.1 (Bruker Biospin); MicroCal VP-ITC (Malvern)
Data analysis	MicroCal ITC-ORIGIN Analysis Software (Malvern); PyMOL Molecular Graphics System Version 2.4 (Schrödinger, LLC); TALOS-N (DOI: 10.1007/s10858-013-9741-y); CcpNMR 2.5 (https://doi.org/10.1007/s10858-016-0060-y); AmberTools21 (https://ambermd.org); matplotlib (http://dx.doi.org/10.1109/MCSE.2007.55); MDAnalysis (https://doi.org/10.1002/jcc.21787); BitQt (https://doi.org/10.1093/bioinformatics/btab595); ARIA/CNS (https://doi.org/10.1093/bioinformatics/19.2.315)

For manuscripts utilizing custom algorithms or software that are central to the research but not yet described in published literature, software must be made available to editors and reviewers. We strongly encourage code deposition in a community repository (e.g. GitHub). See the Nature Portfolio guidelines for submitting code & software for further information.

Data

Policy information about availability of data

All manuscripts must include a data availability statement. This statement should provide the following information, where applicable:

- Accession codes, unique identifiers, or web links for publicly available datasets
- A description of any restrictions on data availability
- For clinical datasets or third party data, please ensure that the statement adheres to our policy

Molecular Dynamic data has been submitted to Zenodo (https://zenodo.org/doi/10.5281/zenodo.10963887); The assignment of FOXO4FH, FOXO4CR3, p53TAD2 ,p531-94 and p53DBD had already been submitted to the BMRB under the accession numbers 50398, 50402, 51125, 51124 and 51753, respectively.

Resonance assignments of FOXO4-LRI was submitted to the BRMB under the accession number 52458. The BMRB files corresponding to Figures 1c-f, 2b-c, 4b-d, 5b, 6c and Supplementary Figures 3b-d, 7e-g have been submitted and are waiting for approval.

Research involving human participants, their data, or biological material

Policy information about studies with human participants or human data. See also policy information about sex, gender (identity/presentation), and sexual orientation and race, ethnicity and racism.

Reporting on sex and gender	N/A
Reporting on race, ethnicity, or other socially relevant groupings	N/A
Population characteristics	N/A
Recruitment	N/A
Ethics oversight	N/A

Note that full information on the approval of the study protocol must also be provided in the manuscript.

Field-specific reporting

Please select the one below that is the best fit for your research. If you are not sure, read the appropriate sections before making your selection.

[x] Life sciences [ ] Behavioural & social sciences [ ] Ecological, evolutionary & environmental sciences

For a reference copy of the document with all sections, see nature.com/documents/nr-reporting-summary-flat.pdf

Life sciences study design

All studies must disclose on these points even when the disclosure is negative.

Sample size	No sample size calculation was performed (in vitro study)
Data exclusions	No data was excluded
Replication	Most ITC measurements were performed three times (technical replicates) and results are presented as mean. Pull-down assays were performed in triplicates. All NMR measurements were performed only once.
Randomization	Not relevant to our study (in vitro study, technical replicates)
Blinding	Not relevant to our study (in vitro study, technical replicates)

Reporting for specific materials, systems and methods

We require information from authors about some types of materials, experimental systems and methods used in many studies. Here, indicate whether each material, system or method listed is relevant to your study. If you are not sure if a list item applies to your research, read the appropriate section before selecting a response.

Materials & experimental systems

n/a	Involved in the study	
[ ]	[x]	Antibodies
[ ]	[x]	Eukaryotic cell lines
[x]	[ ]	Palaeontology and archaeology
[x]	[ ]	Animals and other organisms
[x]	[ ]	Clinical data
[x]	[ ]	Dual use research of concern
[x]	[ ]	Plants

Methods

n/a	Involved in the study	
[x]	[ ]	ChIP-seq
[x]	[ ]	Flow cytometry
[x]	[ ]	MRI-based neuroimaging

Antibodies

Antibodies used	Pp53 polyclonal antibody (rabbit, 1:3000, Cat # 10442-1-AP, Lot # 00135720, Proteintech); goat-anti rabbit HRP conjugate (goat, 1:10000, Cat # 1858415, Lot # HG106488, Pierce)

Validation	The suppliers validated the used antibodies.

Eukaryotic cell lines

Policy information about cell lines and Sex and Gender in Research

Cell line source(s)	p53 mutant human TERT-immortalized retinal pigment epithelial 1 (RPE1) cells (RPE1 TP53−/−; denoted RPE-1 p53KO) cells were kindly provided by Joanna Loizou (CeMM Research Center for Molecular Medicine of the Austrian Academy of Sciences, Vienna, Austria)
Authentication	The RPE-1 cell lines were re-validated by STR profiling.
Mycoplasma contamination	The cell line was tested negative for mycoplasma contamination.
Commonly misidentified lines (See ICLAC register)	N/A (RPE-1 cells are not among the misidentified cell lines)

Plants

Seed stocks	N/A
Novel plant genotypes	N/A
Authentication	N/A
